# Modified hydrothermal method for synthesizing titanium dioxide-decorated multiwalled carbon nanotube nanocomposites for the solar-driven photocatalytic degradation of dyes†

**DOI:** 10.1039/d4ra05899b

**Published:** 2024-10-25

**Authors:** Nhu-Bao Trinh, Thu Anh Nguyen, Sy Van Vu, Hong-Gam Thi Vo, Tien Nu Hoang Lo, In Park, Khuong Quoc Vo

**Affiliations:** a Faculty of Chemistry, Ho Chi Minh City University of Science, Vietnam National University, Ho Chi Minh City 227 Nguyen Van Cu Street, Ward 4, District 5 Ho Chi Minh City 70000 Vietnam vqkhuong@hcmus.edu.vn; b Vietnam National University Ho Chi Minh City Vietnam; c Research Institute of Clean Manufacturing System, Korea Institute of Industrial Technology (KITECH) 89 Yangdaegiro-gil, Ipjang-myeon Cheonan 31056 South Korea; d KITECH School, University of Science and Technology (UST) 176 Gajeong-dong, Yuseong-gu Daejeon 34113 South Korea

## Abstract

This study aimed to address the issue of rapid electron–hole recombination in photocatalysis by exploiting multi-phase TiO_2_ decorated on multiwalled carbon nanotubes (MWCNTs) to improve the photocatalytic degradation of dyes. A simple and eco-friendly one-pot method was utilized to create the TiO_2_/MWCNT nanostructure using glucose as both a structure-directing agent and a carbon source without requiring any prior covalent or non-covalent functionalization of the MWCNTs at 160 °C. Furthermore, it was found that the average width of the nanocomposites changed from 20 ± 1 and 42 ± 2 nm to 56 ± 3 nm, corresponding to MWCNT contents of 1.0, 2.0, and 3.0 (wt%), respectively. Specifically, TiO_2_/MWCNTs with a low content of MWCNTs demonstrated enhanced performance for the photocatalytic degradation of dyes, with the bandgap of the nanocomposites decreasing to 2.5 eV with 1.0% MWCNTs and 2.4 eV with 2.0% MWCNTs. The TiO_2_/MWCNT-1.0 catalyst demonstrated high photocatalytic efficiency for methylene blue (MB) degradation with a rate constant of 0.0051 min^−1^. TiO_2_/MWCNT-2.0 was more effective for rhodamine (RhB) degradation than pristine TiO_2_, with a rate constant of 0.0065 min^−1^ within 120 min of solar-light exposure. This novel modified approach can be used to synthesize nanocomposites simply and is potentially feasible for efficient dye degradation and beyond, offering a promising solution for water-pollution treatment.

## Abstract 2

Address the issue of rapid electron-hole recombination in photocatalysis by exploiting multi-phase TiO_2_ decorated on multiwalled carbon nanotubes (MWCNTs) to improve the photocatalytic degradation of dyes.

## Graphical abstract

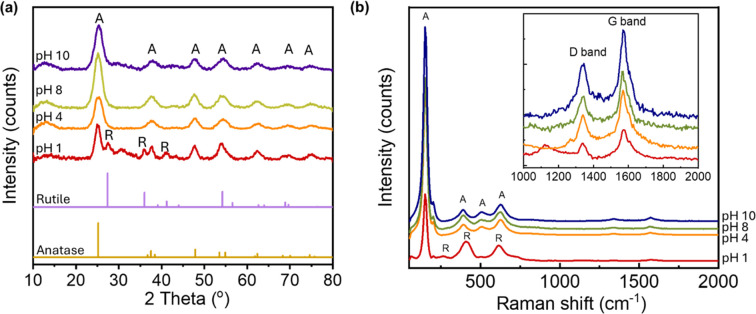

## 1 Introduction

One of the most pressing environmental issues in modern times is the contamination of natural water systems caused by many factors, such as population growth, urbanization,^1^ and rapid industrialization.^2^ Among these, industrial activities—particularly in the textile, food processing, dyeing, paper, and dye manufacturing sectors—are significant sources of water pollution.^3^ The pollutants can dissolve in water, remain suspended,^4^ or settle on sediments,^5^ thereby degrading the water quality.^6^ In particular, organic dyes released from many industries are dominant health threats to living organisms and human beings.^7^ RhB is one of the reddish violet dyes that can have harmful effects on human health, causing eye and skin irritation and even malfunction of the respiratory system.^8^ Another dye is MB, which is toxic and non-biodegradable, and it can induce dizziness, headache, and nausea.^9^ Therefore, RhB and MB dye removal in the aqueous environment is fundamentally necessary.

Titanium dioxide (TiO_2_) is widely regarded as the leading photocatalyst for breaking down dyes in wastewater because of its low cost and toxicity, high photoactivity^10^ and chemical stability, and environmental friendliness.^11^ However, the high band gap of TiO_2_ (3.2 eV for anatase and 3.0 eV for rutile phase) decreases the photocatalyst efficiency in visible light. Besides, a major challenge in photocatalyst applications is the rapid recombination of photoexcited electrons and holes, which diminishes the quantum efficiency and photocatalytic activity.^12^ Much research has been conducted to solve this problem by integrating it with conductive organic materials, such as graphene (Gr), graphene oxide (GO), and carbon nanotubes (CNTs), which could greatly enhance the photocatalytic performance of TiO_2_ due to accelerated electron transfer and improved charge-separation efficiency.^13^

Carbon nanotubes (CNTs) have garnered considerable attention due to their high conductivity, large specific surface area, and porous structure, which can enhance the overall photocatalytic effectiveness.^14^ At the TiO_2_–CNT interface, a photoexcited electron (e^−^) can be transferred to the CNT, which has a lower Fermi level.^15^ Therefore, electrons *ca* be persistently accepted on CNTs, and the recombination rate of photoexcited electron–hole pairs could be decreased.^16^ Furthermore, it was reported that incorporating CNTs into a composite with TiO_2_ provided ample active sites on the surface, significantly enhancing the photodegradation rate of dyes.^17^ Thus, TiO_2_/CNTs nanocomposites are promising materials for attaining high photocatalytic activity and have consequently attracted significant attention in different areas, such as wastewater treatment,^18^ water splitting, photocatalytic reduction of CO_2_,^19^ sensor devices, and sensing applications.^20^ Mohamed Shaban *et al.* found that a composite of titanium dioxide nanorods on carbon nanotubes (TiO_2_NRs/CNTs) could halve the irradiation time required to degrade MB dye compared to pure TiO_2_ nanorods (NRs) under sunlight.^21^ Shaari *et al.* examined rare earth elements Ce-doped CNT–TiO_2_ and recorded a significant degradation of about 94% of phenol at a concentration of 50 mg L^−1^ over 3 h.^22^ Moreover, a combination of the two phases of TiO_2_, anatase and rutile, is known to have synergistic effects, resulting in remarkably improved photocatalytic activity compared to the individual pure phases.^23^ Though not yet fully understood, this effect may involve the migration of photoexcited charges between the two phases, leading to enhanced charge separation.^24^ Although numerous studies have been conducted on CNT–TiO_2_ nanocomposites to improve photocatalytic activity, multi-phase compositions of TiO_2_ have not received much attention.

In this research, we investigated the synergistic effects between the multi-phase of anatase-rutile TiO_2_ and MWCNTs (multiwalled CNTs). This addresses a significant challenge in TiO_2_ photocatalysis, involving the rapid recombination of electrons and holes and decreased band gap to absorb visible light efficiently. Here, TiO_2_/MWCNTs nanocomposites were synthesized by a novel modified process based on a hydrothermal method, and the phase composition showed a dependence on the pH conditions. Moreover, we also adapted a simple, eco-friendly one-pot method to produce a TiO_2_/MWCNT nanostructure utilizing glucose as an adhesion bridge between the TiO_2_ and MWCNTs. The characterization of the TiO_2_/CNT nanocomposites at different pH conditions exhibited the presence of multiple phases at pH 1.0 and the predominance of the anatase phase at higher pH values. Besides, the percentage of MWCNTs had an impact on the nanomaterial with the bandgap decreasing to 2.5 eV with 1.0% MWCNTs and 2.4 eV with 2.0% MWCNTs compared to pure TiO_2_ with a band gap of 3.1 eV, which were remarkably lower than reported in previous studies.^14^ From this,^25^ it can be seen that the TiO_2_/MWCNT catalyst demonstrated higher photocatalytic efficiency for the photodegradation of MB and RhB. These findings indicated that the as-developed TiO_2_/MWCNTs nanocomposites offers significantly enhance photocatalytic performance compared to TiO_2_, and is feasible for application in environmental treatment.

## 2 Experimental

### 2.1. Chemicals and materials

Titanium isobutoxide (TTIB, ≥99.0%), multiwalled carbon nanotubes (MWCNTs, ≥80% as carbon nanotubes), d-glucose (C_6_H_12_O_6_, ≥99.0%), and hydrochloric acid (HCl, 37%) were purchased from Sigma-Aldrich Chemie GmbH (Taufkirchen, Germany). Sulfuric acid (H_2_SO_4_, 95.0–98.0%), methylene blue (MB, ≥99.0%), and rhodamine B (RhB, ≥99.0%) were obtained from Xilong Scientific Co., Ltd (Jiangsu, China). Deionized (DI) water was used to prepare all the solutions. All the reagents were directly used without further purification.

### 2.2. Synthesis of TiO_2_/MWCNTs nanocomposite by a modified hydrothermal method

In the typical synthesis for the TiO_2_/MWCNTs 1.0% sample as representative, 37 mL of glucose 1.95% (w/v), 3.0 mL of hydrochloric acid 36.0% (v/v), and 0.06 g of MWCNTs were respectively added into z glass beaker under stirring at 600 rpm. Subsequently, 6.0 mL of titanium isobutoxide (TTIB) was added dropwise into the mixture and stirred for 30 min. Then, the mixture was poured in to a Teflon tube (90 mL) lined with a hydrothermal steel autoclave and heated at 160 °C for 6 h. Afterward, the mixture was naturally cooled to room temperature and centrifuged at 6000 rpm for 10 min. The obtained precipitant was washed three times with DI water to remove the undesired byproducts and the residual precursors. The products were finally dried at 60 °C over 480 min to obtain TiO_2_/MWCNTs nanocomposites. Subsequently, the TiO_2_/MWCNTs nanocomposites were synthesized under different pH conditions (ranging from 1, 4, 8, to 10) and adjusted using 1.0 M HCl and 1.0 M NaOH aqueous solutions. The MWCNTs contents were varied and examined at 1.0, 2.0, and 3.0 wt%, namely TiO_2_/MWCNTs-1.0, TiO_2_/MWCNTs-2.0, and TiO_2_/MWCNTs-3.0, respectively.

### 2.3. Investigation of the photocatalytic activity of the TiO_2_/MWCNTs nanocomposite

The performances for the photodegradation of dyes (RhB and MB) were studied with the TiO_2_/MWCNTs catalyst using a solar-light simulator Moritex MME-250 (Moritex Corporation) and UV light. Briefly, 60.0 mg of TiO_2_/MWCNTs was added into 100 mL of a 5.0 mg L^−1^ aqueous dye solution. Subsequently, the mixture was stirred at 300 rpm in the dark for 30 min to attain the adsorption/desorption equilibrium and then irradiated to ignite the dye photodegradation reaction. After each interval time, a 5.0 mL solution was extracted and centrifuged at 6000 rpm to remove the catalyst. The dye concentration at the interval time was determined by UV-vis spectroscopy (at *λ*_max(MB)_ = 664 nm, *λ*_max(RhB)_ = 554 nm). The photodegradation efficiency was calculated using eqn (1):1
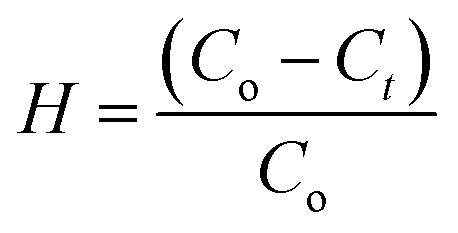
where *C*_*t*_ is the concentration of the organic dyes studied at the interval times, *C*_o_ is the initial concentration of dyes, and *H* is the degradation efficiency.

The degradation rate constant was determined based on the first-order kinetics using eqn (2):2
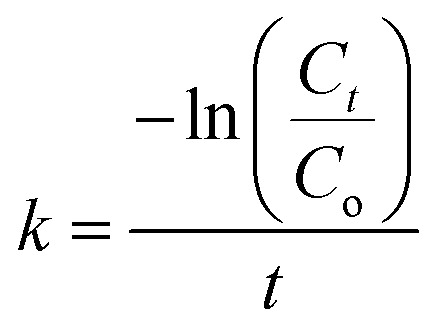


For the trapping experiment tests, potassium iodide (KI), dimethyl sulfoxide (DMSO), benzoquinone (BQ), and isopropyl alcohol (IPA) were used as trapping reagents to identify the role of different reactive species, specifically photogenerated holes (h^+^), electrons (e^−^), superoxide radicals (˙O^2−^), and hydroxyl radicals (˙OH), respectively. The dye solution was prepared with 0.01 M of each trapping reagent, and the experimental procedure was conducted similarly to the dye degradation experiments, using a catalyst dosage of 0.4 g L^−1^. The used TiO_2_/MWCNTs were re-collected, rinsed with distilled water, and air-dried under ambient conditions.

### 2.4. Characterization of the photocatalysts

Ultraviolet-visible (UV-vis) spectra of the dye solution and diffuse reflectance spectra (DRS) of the solid state of TiO_2_/MWCNTs were recorded using a Jasco V-670 UV-vis spectrophotometer, equipped with a Jasco-ARN 731 accessory in the wavelength range from 250 to 850 nm. UV-vis measurements were conducted using a quartz cuvette and blank sample for further comparison. For analyzing the TiO_2_/CNT nanocomposites, the sample was evenly spread on a quartz film to avoid inconsistent signal measurements. The flat band potentials of the TiO_2_/MWCNTs-1.0 and TiO_2_/MWCNTs-2.0 nanocomposites were determined using Mott–Schottky (M–S) plots. In such plots, the linear portion of the plot reflects the depletion region of carriers in the space charge region, as defined by eqn (3):^16^3
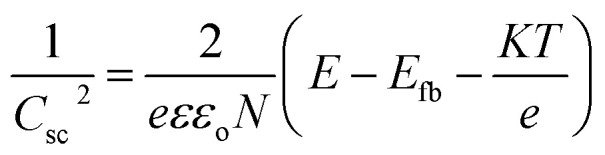
where *C*_sc_ is the specific capacity, *ε* is the relative dielectric constant of the material, *ε*_o_ is the permittivity for vacuum space, *N* is the donor density for the semiconductor, *E* is the potential application, *E*_fb_ is the flat band potential, *K* is the Boltzmann constant, *T* is the absolute temperature, and *e* is the electronic charge.

FE-SEM (JEOL JSM-7600F, USA) was used to analyze the morphology of the TiO_2_/MWCNTs, utilizing a Quanta FEG 250 instrument at an accelerating voltage of 10.0 kV. The elemental composition on the surface of TiO_2_/MWCNTs was also analyzed by energy-dispersive X-ray spectroscopy (EDS) using an Oxford Instruments 50 mm^2^ X-max (UK) detector and a Gatan mono system (Gatan, UK). The TiO_2_/MWCNTs were further examined by transmission electron microscopy with an accelerating voltage of 200 kV. The crystalline structure and surface morphology of the heterojunction nanocomposite photocatalysts were analyzed through high-resolution transmission electron microscopy (HR-TEM) using a JEOL ARM 200F instrument (JEOL Ltd, Japan). The elemental distribution in the TiO_2_/MWCNT nanomaterials was assessed by TEM mapping with a JEOL JEM-2100 instrument (JEOL Ltd, Tokyo, Japan). For TEM analysis, a small portion of the sample was dispersed in ethanol *via* sonication and then placed onto a copper grid coated with a lacy carbon film. Moreover, the crystal structure of the materials was examined using X-ray diffraction (D8 Advance, Bruker, Germany), with analysis angles ranging from 30° to 80° and an angle change rate of 0.02° per sec.

The interactions between the functional groups on the surface of MWCNTs and TiO_2_ were investigated based on their Fourier transform infrared (FT-IR) spectra over the range 4000–400 cm^−1^ using an FT/IR-6600 type A system (Jasco, Japan). The chemical composition at the surface of the samples was determined by X-ray photoelectron spectrometry (XPS; Thermo Scientific, Waltham, MA, USA) with a monochromatic Al Kα X-ray source at a photon energy of 1486.7 eV. The textural properties of the prepared materials were measured using a Tristar II Plus 3030 instrument (Micromeritics, USA). BET surface analysis was used to determine the surface area using a partial pressure (*P*/*P*_o_) range of 0.01–0.45. The total pore volume was calculated at a single-point adsorption *P*/*P*_o_ of 0.950, and the pore-size distribution was assessed using the Barrett–Joyner–Halenda (BJH) porosity model. Raman spectra were evaluated using an XploRa Plus confocal Raman microscope (Horiba SAS, Longjumeau, France) with an excited laser source of 532 nm, power of 25 mW, and 50× objective lens. The catalytic activity of the materials was studied using a sunlight simulator system (MORITEX MME-250-220, Metal Halide Light Source, 250 W).

The photoelectrochemical (PEC) properties of the TiO_2_/MWCNTs were evaluated using a Gamry Interface 1010T system. In detail, 5.0 mg TiO_2_/MWCNTs were dispersed in 2.0 mL isopropanol, and then 0.5 mL of solution was dropped onto a fluorine-doped tin oxide (FTO) electrode (1.0 × 1.0 cm) and dried at 100 °C for 2 h. The measurements were performed using a three-electrode PEC system, with Ag/AgCl as the reference electrode, Pt sheet as the counter electrode, and TiO_2_/MWCNTs-coated FTO electrode as the working electrode (WE) in 0.5 M Na_2_SO_4_ solution. The transient photocurrent responses were measured using the chronoamperometry method to determine the carrier mobility of the materials. Mott–Schottky plots were obtained to determine the flat band potential of the materials,^16^ measured in the dark conditions in the potential range from −0.8 to 0.5 V and a frequency of 10 Hz. Electrochemical impedance spectroscopy (EIS) was performed in the range from 0.1 Hz to 10 kHz.

### 2.5. Cyclic performance using TiO_2_/MWCNTs

The stability of catalysts plays a crucial role in assessing their practicality and cost-efficiency. To evaluate the operational stability, the TiO_2_/MWCNTs photocatalyst was repeatedly tested for RhB degradation over several cycles under identical conditions (0.4 g L^−1^ catalyst concentration, 120 min of irradiation, 5.0 mg L^−1^ dye solution, and neutral pH). After each cycle, the catalyst was washed with distilled water, centrifuged at 6000 rpm, and dried for 6 h at 80 °C.

## 3 Results and discussion

### 3.1. Synthesis of TiO_2_/MWCNTs nanocomposites

The TiO_2_/MWCNTs nanocomposites were initiated with the hydrothermal process involving strong Lewis acid TTIB precursors in the presence of hydrochloric acid (HCl) and glucose diluted with deionized water (DI). When the TTIB hydrolyzes with water, Ti^4+^ increases from +4 to +6, thus forming Ti–O bonds. Under high pressure and temperature, these six-fold structural units form octahedra that eventually precipitate into crystals. Corner- and edge-sharing occur as the octahedra aggregate during condensation reactions and form TiO_2_.^26^ The chemical reactions involved in the hydrolysis (eqn (4)) and dehydration or condensation (eqn (5) and (6)) during the hydrothermal synthesis process can be described as follows:

4 Ti(OR)_4_ + 4H_2_O → Ti(OH)_4_ + 4ROH

5 Ti(OH)_4_ + Ti(OH)_4_ → 2TiO_2_ + 4H_2_O

6 Ti(OH)_4_ + Ti(OR)_4_ → 2TiO_2_ + 4ROH

The overall reaction is:

7 Ti(OR)_4_ + 4H_2_O → 2TiO_2_ + 4ROH

The one-pot synthesis of TiO_2_/MWCNTs was achieved using glucose as a structure-directing agent and a carbon source without requiring any prior covalent or non-covalent functionalization of MWCNTs. Using glucose to facilitate the formation of TiO_2_/MWCNTs, it was reported that the hydrothermal process of glucose can produce some aromatic compounds with multiple hydroxyl groups, which act as a bridge to connect TiO_2_ and MWCNTs. The phenyl ring of these aromatic compounds attach to the graphitic surface of CNTs through π–π stacking, while its hydroxyl group directly interact with the TTIB precursors.^27^

### 3.2. Effect of pH

**Fig. 1:**
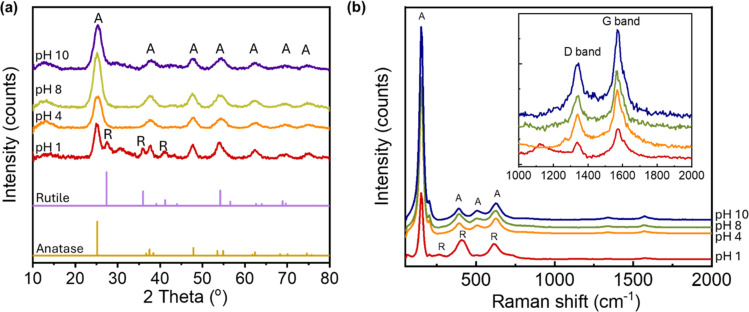
(a) XRD patterns and (b) Raman spectra of TiO_2_/MWCNTs-2.0 synthesized at different pH conditions, including pH 1, 4, 8, 10.

TiO_2_/MWCNTs-2.0% was prepared at different pH values (1, 4, 8, and 10) to evaluate the effect of the pH conditions on the TiO_2_/MWCNTs nanocomposites formation. XRD patterns and Raman spectra were employed to determine the crystalline structure of the samples, as shown in Fig. 1. The XRD pattern (Fig. 1(a)) of TiO_2_/MWCNTs-2.0% at pH 1 displayed all the diffraction peaks of the tetragonal anatase TiO_2_ (JCPDS No. 21-1272) and other peaks at 2-theta of 27.4°, 35.9°, and 41.1°, which were related to the (110), (101), and (111) facets of the rutile TiO_2_ (JCPDS No. 21-1276).^28^ At higher pH values, only diffraction peaks characteristic of the anatase phase were observed, and the intensity of the anatase TiO_2_ (101) diffraction peak at 25.2° increased as the pH increased from 4 to 8, but decreased at pH 10. This indicates that the pH influences the phase transition from rutile to anatase and affects the crystallinity of TiO_2_ during the hydrothermal process. Additionally, the crystal size was estimated using Scherrer's equation, as given in eqn (8):^29^8
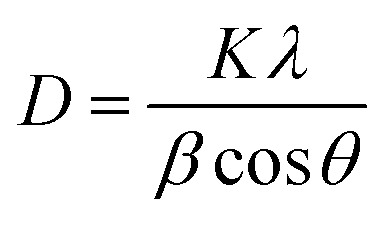
where *D* represents the crystalline size (nm), *K* is 0.9 (Scherrer's constant), *λ* is 0.15406 nm (the wavelength of the X-ray source—CuKα), *β* is the full width at half maximum FWHM (in radians), and *θ* is the Bragg-diffraction angle (peak position in radians) (Table 1).

**Table 1:** Variation in crystalline size with varying the pH condition as determined by Scherrer's equation

Sample	2 theta (°)	FWHM (°)	*D* (nm)
pH 1	25.12	1.56	5.4
pH 4	25.30	2.19	3.9
pH 8	25.19	2.18	3.9
pH 10	25.35	2.25	3.8

### 3.3. Crystalline structure and composition

Further evidence for the crystalline structure of TiO_2_/MWCNTs was obtained from Raman spectroscopy (Fig. 1(b)), which recorded the characteristic vibrations of TiO_2_. At pH 1, the spectrum revealed the formation of a rutile and anatase phase mixture, as indicated by the peak at 150 cm^−1^ assigned to the symmetries of the E_g_ mode of anatase, and two stretching peaks at 409 and 618 cm^−1^ corresponding to the symmetries of the E_g_ and A_1g_ of rutile TiO_2_. As the pH increased, there were only Raman active modes of anatase TiO_2_ shown by peaks at 150 cm^−1^ (E_g_), 390 cm^−1^ (B_1g_), 508 cm^−1^ (A_1g_), and 624 cm^−1^ (E_g_).^30^ Additionally, the Raman spectra showed signals at 1340 and 1574 cm^−1^, corresponding to the D and G bands of MWCNTs, respectively, indicating that the hydrothermal process preserved the structure of the CNTs. The ratio of the D and G bands intensity (*I*_D_/*I*_G_) in a CNT sample is indicative of the presence of structural defects and sp^3^-hybridized carbon atoms, providing insights into the level of sidewall functionalization.^31^ The *I*_D_/*I*_G_ ratio varied from 0.52, 0.57, and 0.58 to 0.60, at pH values of 0, 4, 8, and 10, respectively. A higher *I*_D_/*I*_G_ ratio was observed at elevated pH values, implying the interaction between the MWCNTs surfaces and TiO_2_ nanoparticles.

The pH value directly affects the dispersion of MWCNTs in water and the process of attaching TiO_2_ to the MWCNTs surface. Multiwalled carbon nanotubes (MWCNTs) comprise non-polar graphene walls, making them poorly dispersed in water. However, when the pH of the solution increases, the number of COOH functional groups on the surface of MWCNTs that deprotonate to form carboxylate anions (COO^−^) also increases. This process enhances the dispersion of MWCNTs in water.^32^ The negative charge positioned on the two oxygen atoms in the carboxylate anion will repel each other, loosening the CNT bundles and helping the CNT have many opportunities for separate distribution, which is beneficial in the process of TiO_2_ agglomeration on the MWCNTs surface.

In the hydrothermal method, the acidity was supposed to be crucial for the hydrolysis of TTIB. Under highly acidic conditions (with a high concentration of H^+^ ions), the hydrolysis of titanium isobutoxide (TTIB) can lead to the formation of protonated groups as intermediate compounds [Ti(OH)_3_(OH_2_)_3_]^+^.^26^ These intermediate compounds will readily bond with OH^−^ groups from other TiO_6_ octahedra. Then, Ti–O–Ti oxygen bridge bonds are formed with water dehydration. To achieve a dense and favorable orientation in the rutile phase, the TiO_6_ octahedra must exhibit a high degree of protonation, making hydrolysis and dehydration reactions effectively catalyzed. Thus, both rutile and anatase phases were obtained at pH 1.0. In contrast, higher pH conditions that lead to the increase in the concentration of OH^−^ ions promote the rapid oxidation of titanium precursors to form Ti(OH)_3+*x*_, resulting in the formation of only the anatase phase.^23^ Following the dehydration reaction, insufficient protonation leads to a face-sharing anatase phase, while adequate protonation results in corner- and edge-sharing in the rutile phase.^26^

**Fig. 2:**
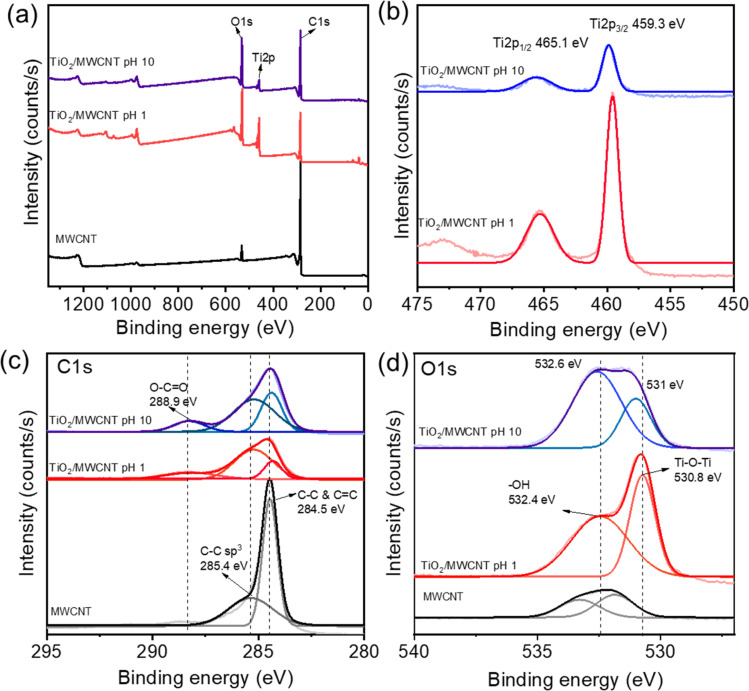
(a) Survey XPS spectra, (b) high-resolution Ti 2p XPS spectra, (c) C 1s XPS spectra, and (d) O 1s XPS spectra of bare MWCNTs, and TiO_2_/MWCNTs 2.0% nanocomposite synthesized at pH 1.0 and pH 10.0.

XPS was next employed to determine the surface components of the TiO_2_/MWCNTs. The XPS spectra of TiO_2_/MWCNTs-2.0 synthesized at pH 1 and 10 are shown in Fig. 2(a). The XPS survey spectra indicated the interaction of TiO_2_ and CNT by the appearance of C, O, and Ti elements in both samples, while pristine MWCNTs only presented O and C atoms. The Ti 2p high-resolution XPS (HR-XPS) spectrum of TiO_2_/MWCNTs-2.0 synthesized at pH 1 (Fig. 2(b)) displayed two peaks at binding energies (BE) of approximately 465.1 and 459.3 eV, corresponding to Ti 2p_3/2_ and Ti 2p_1/2_ splitting of the Ti^4+^ state.^33^ The analysis of the Ti-2p peaks showed that the samples contained Ti^4+^. For the sample synthesized at pH 10, these peak positions were shifted to around 465.3 and 459.6 eV for the Ti^4+^ state, and these intensities were significantly decreased. Compared to standard values, the higher binding energies of the Ti 2p_3/2_ and Ti 2p_1/2_ peaks were attributed to the formation of Ti–C bonds.^34^ Moreover, the Ti 2p high-resolution XPS (HR-XPS) spectra of the TiO_2_ sample (Fig. S1(b)†) showed two peaks at binding energies (BE) of around 464.7 and 459.0 eV, corresponding to the splitting of Ti 2p_3/2_ and Ti 2p_1/2_ in the Ti^4+^ state. The Ti 2p HR-XPS spectra of TiO_2_/MWCNTs exhibited a slight shift to higher binding energies compared to TiO_2_, indicating the presence of lattice bonding in the form of Ti–O–C. This shift in binding energy was also attributed to lattice distortion, further confirming the interaction between TiO_2_ and carbon.^35^ The HR-XPS spectra of C 1s (Fig. 2(c)) further provided more evidence for the presence of Ti–C bonds. For pristine MWCNTs, there was a sharp peak at 284.5 eV corresponding to C–C and C=C sp^2^ hybridization and a weak peak around 285.4 eV related to C–C sp^3^ hybridization.^36^ In the case of TiO_2_/MWCNTs 2.0% synthesized at pH 1.0 and 10, these peaks were shifted to a lower binding energy of 284.3 eV. This shift was associated with the formation of Ti–C bonds in the nanocomposites.^37^ Meanwhile, other peaks in the nanocomposites were observed, centered at a BE of 288.9 eV, which were attributed to C sp^3^ and C=O species bonded to oxygen.^15^ These functional groups in the functionalized CNTs undergo an esterification reaction with the –OH groups of the Ti precursor, forming covalent bonds, such as C–O–Ti or O=C–O–Ti. The HR-XPS O 1s spectrum of TiO_2_/MWCNTs-2.0 synthesized at pH 1.0 showed two deconvoluted peaks at approximately 530.8 and 532.6 eV, corresponding to lattice oxygen (O_2_^2−^) in anatase Ti–O–Ti and surface hydroxyl groups (–OH), respectively.^38^ For TiO_2_/MWCNTs-2.0 synthesized at pH 10, the lattice oxygen peak decreased, and the non-lattice peak significantly increased, indicating the formation of more oxygen defects in the crystal structure when synthesized in alkaline conditions.

**Fig. 3:**
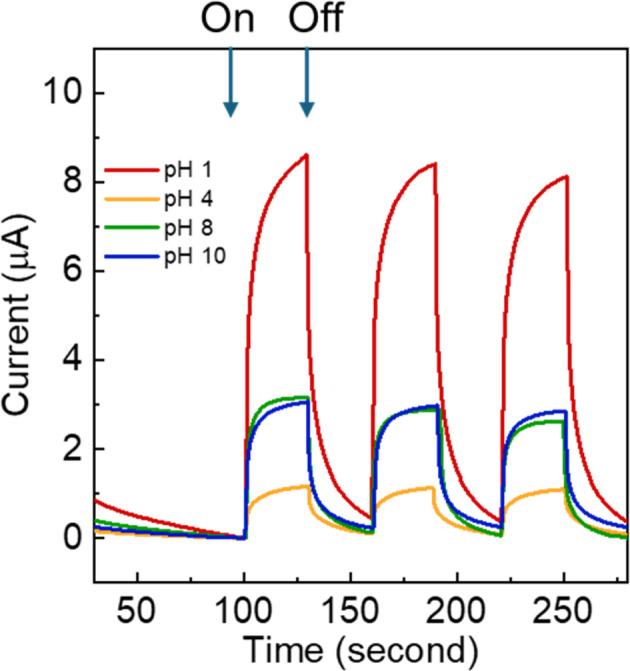
Transient photocurrent responses of TiO_2_/MWCNT nanocomposites synthesized under different pH conditions (pH 0, 4, 8, and 10).

Next, photoelectrochemical (PEC) measurements were employed to investigate the photocatalyst performance of the nanocomposites. The photocurrent response of TiO_2_/MWCNTs nanocomposites synthesized at different pH values (1, 4, 8, and 10) was recorded over three on–off cycles of light irradiation. As shown in Fig. 3, all four samples exhibited reversible photocurrent responses and high reproducibility under light-on and light-off conditions, indicating the efficient separation and transfer of photogenerated electron–hole pairs.^39^ The TiO_2_/MWCNTs synthesized at pH 1 demonstrated the highest photocurrent, approximately 9 μA cm^−2^ (curve red), while the photocurrent significantly decreased for the samples synthesized at pH 4, 8, and 10. These results suggest that the mixture of anatase and rutile TiO_2_ phases can enhance the photoelectron density of the TiO_2_ photocatalyst rather than the pure anatase crystal phase, thus enabling a higher photocatalyst performance.^40^ This proved that the TiO_2_/MWCNTs nanocomposite synthesized in an acidic environment exhibited higher photocatalyst performance.

### 3.4. Effect of the MWCNT contents

**Fig. 4:**
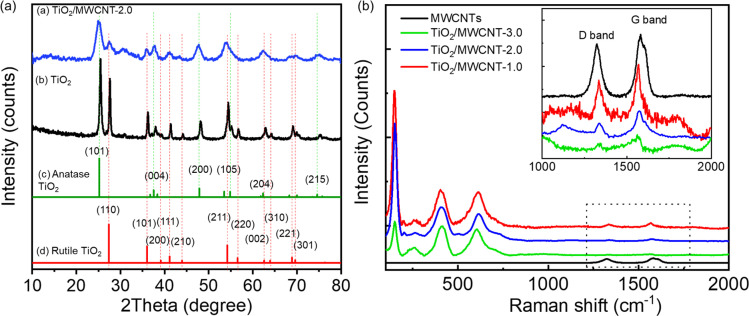
(a) XRD patterns of (a) TiO_2_/MWCNTs-2.0 nanocomposite, (b) pure TiO_2_, and standard patterns of (c) anatase TiO_2_ and (d) rutile TiO_2_. (b) Raman spectra of TiO_2_/MWCNTs synthesized at different percentages of MWCNTs, including 1.0, 2.0, and 3.0 wt%.

The XRD patterns of pristine TiO_2_ and TiO_2_/MWCNTs-2.0 nanocomposites (Fig. 4) indicated the presence of both rutile and anatase phases of TiO_2_ in both samples.^41^ The absence of a MWCNTs peak in the TiO_2_/MWCNTs-2.0 nanocomposite pattern was due to the prominent TiO_2_ peak at 2*θ* = 25.3°, which might overlap the MWCNTs peak at 2*θ* = 25.9°. Compared to the pristine TiO_2_ pattern, a significant decrease in the intensity of all peaks in the TiO_2_/MWCNTs nanocomposite could be observed. Besides, the anatase TiO_2_ (101) peak at 2*θ* = 25.3° and the rutile (110) peak at 2*θ* = 27.4° became broadened, indicating that the addition of MWCNTs reduced the crystallinity of TiO_2._ Raman spectra were collected for the MWCNTs and TiO_2_/MWCNTs nanocomposite catalysts with varying weight percentages (1.0, 2.0, 3.0 wt%) of MWCNTs, as illustrated in Fig. 4(b). The results showed that all the samples contained a mixture of rutile and anatase phases. This was evident from the presence of peaks at 150 cm^−1^ associated with the E_g_ mode of anatase and peaks at 409 and 618 cm^−1^ corresponding to the E_g_ and A_1g_ symmetries of rutile TiO_2_. Compared to the MWCNTs, the D- and G-band signals of the TiO_2_/MWCNTs were reduced, indicating a decrease in amorphous carbon and an increase in the crystallinity of the samples.^16^

**Fig. 5:**
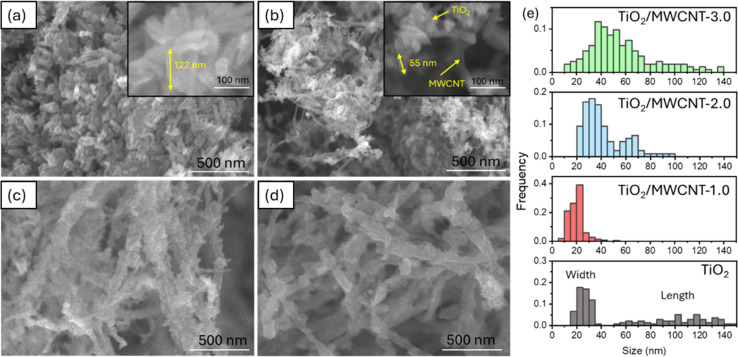
SEM images of (a) TiO_2_ without MWCNTs, (b) TiO_2_/MWCNTs-1.0, (c) TiO_2_/MWCNTs-2.0, and (d) TiO_2_/MWCNTs-3.0 synthesized by the hydrothermal method. (e) The corresponding size distribution graph of the TiO_2_/MWCNT nanocomposite width, synthesized with MWCNT contents of 0, 1, 2, and 3%.

The morphologies of the TiO_2_/MWCNTs nanocomposites synthesized with MWCNT contents of 0, 1.0, 2.0, and 3.0 wt% were characterized by SEM images (Fig. 5). The addition of MWCNTs significantly impacted the morphologies of the TiO_2_ nanoparticles. The sample synthesized without MWCNTs exhibited rod shapes with an aspect ratio of 4 and an average length of 105 ± 3 nm (Fig. 5(a)). In contrast, the TiO_2_/MWCNTs nanocomposites featured smaller TiO_2_ nanoparticles, and this shape became more unclear as the MWCNTs content increased. The TiO_2_/MWCNTs-1.0 nanocomposite (Fig. 5(b)) exhibit the aggregation of smaller TiO_2_ nanorods with an aspect ratio of 2.6 and a length of 18 ± 1 nm. These nanorods formed irregular clusters attached to the MWCNTs. As the MWCNT contents increase from 1.0% to 3.0%, the TiO_2_ layer exhibited a high degree of dispersion that formed a dense and continuous layer around the MWCNTs due to the self-agglomeration and aggregation of small particles (Fig. 5(c and d)). It could be observed that the increase in the average width of MWCNTs from 20 ± 1 and 42 ± 2 nm to 56 ± 3 nm corresponded to the MWCNT content of 1.0, 2.0, and 3.0 wt%, respectively (Fig. 5(e)). Here, the MWCNTs provide a scaffold that supports the attachment and growth of TiO_2_, thus enhancing the surface area and structural integrity.

**Fig. 6:**
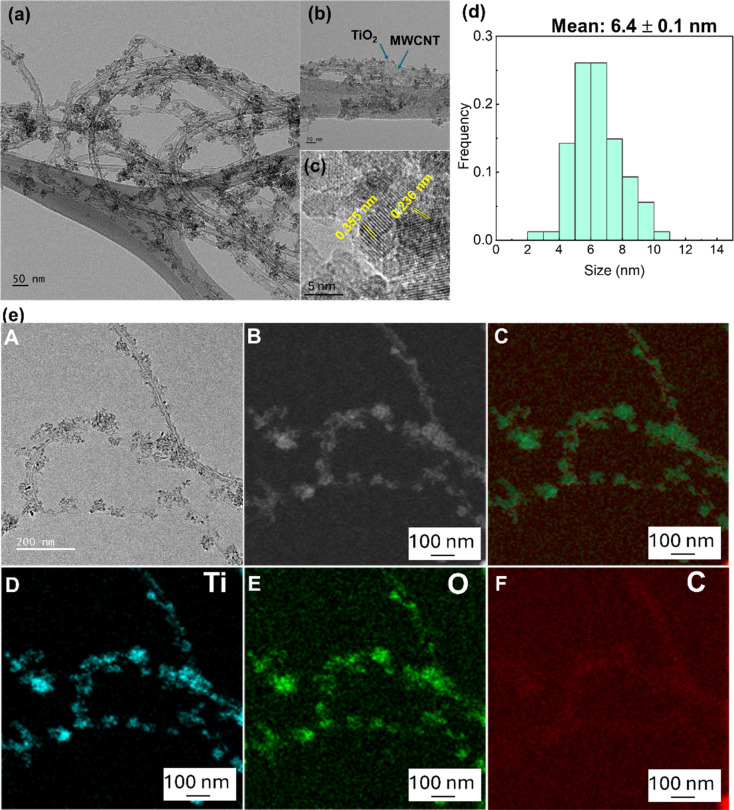
(a) TEM image, scale bar 50 nm. (b) High-magnification TEM image, scale bar 20 nm. (c) HR-TEM image of TiO_2_/MWCNT-2.0 nanocomposites. (d) Size-distribution histogram of TiO_2_ nanoparticles on MWCNTs. (e) TEM-EDS elemental mapping for (D) titanium, (E) oxygen, and (F) carbon.

TEM and HR-TEM images of the TiO_2_/MWCNTs-2.0 nanocomposites were analyzed to confirm the structure of the nanocomposites (Fig. 6(a–c)). These images revealed that small TiO_2_ nanoparticles were uniformly distributed on the surface of the MWCNTs. Fig. 6(d) shows the size-distribution histogram of TiO_2_ nanoparticles, indicating that they were monodispersed with an average size of 6.4 ± 0.1 nm, distributed on the MWCNTs with an average diameter of 17.5 ± 3.0 nm. The HR-TEM image (Fig. 6(c)) displays the crystal lattice fringes with spacings of 0.35 and 0.235 nm, corresponding to the (101) and (001) lattice planes in anatase TiO_2_ decorated on the MWCNTs. Besides, the STEM-EDS mapping images of the TiO_2_/MWCNTs-2.0 nanocomposites (Fig. 6(e)) further demonstrated that TiO_2_ nanocrystals were uniformly distributed around the surface of MWCNTs. The mapping results for titanium (Ti) and oxygen (O) indicated that these elements were evenly distributed on the surface of the CNT fibers. This suggests the successful decoration of TiO_2_ particles on the MWCNTs surface.^42^ Moreover, the carbon (C) element mapping revealed an uneven distribution, further supporting that TiO_2_ particles effectively surrounded the MWCNTs. Additionally, the uneven C distribution indicated the dense attachment of TiO_2_ nanoparticles on the CNT fibers, as the carbon signal was partially obscured by the TiO_2_ layer.^42^

The TiO_2_/MWCNTs-1.0 and TiO_2_/MWCNTs-2.0 nanocomposites were analyzed for determining their elemental composition by X-ray energy-dispersive spectroscopy (EDS) (Fig. S2†). The mass percentages and elements in the corresponding Table S1† also show that the TiO_2_/MWCNTs materials were initially successfully synthesized. The obtained results showed peaks appearing at the 4.6 and 5.0 keV potential regions, which were typical for the Ti element. In addition, peaks appeared at positions 0.2 to 0.6 keV attributed to C and O elements.^43^ The mass percentages and elements presented in the table show that TiO_2_–MWCNTs materials were successfully synthesized with the percentage weight of carbon in TiO_2_/CNT-1.0% and 2.0% at 14.06% and 39.66%, respectively (Table S1†). Besides, the EDS spectrum also presents some peaks of Cl, Si, and S at 2.6, 1.8, and 2.3 keV, respectively.^43^ However, these peak intensities were low compared to the significant elements, possibly because the sample was still mixed with some reactants remaining after the reaction. Fig. S3 (ESI†) presents the electrochemical impedance spectroscopy (EIS) results, illustrating the electron transportation between the electrode interface of TiO_2_/MWCNTs with different MWCNT contents and electrodes, showing that the nanocomposite 2.0% MWCNTs possessed a smaller resistance and faster electron transport with a smaller radius arc in the Nyquist plots.^44^

**Fig. 7:**
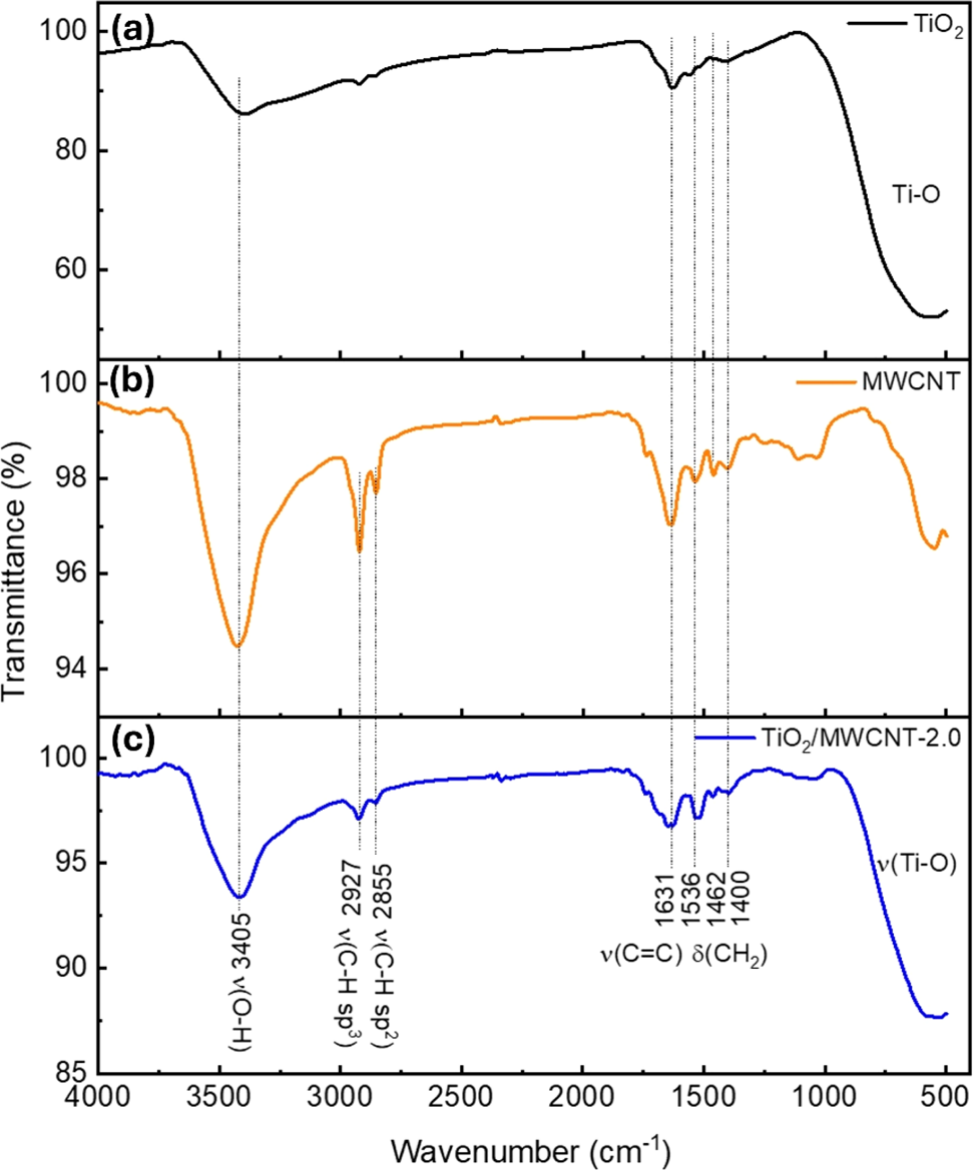
FTIR spectra of (a) bare MWCNTs, (b) pristine TiO_2_, and (c) TiO_2_/MWCNTs-2.0 nanocomposites.

Fourier transform infrared (FTIR) spectroscopy was next used to analyze the chemical bonding and functional groups of the nanostructured TiO_2_/MWCNTs. A series of spectra of MWCNTs, TiO_2_, and TiO_2_–MWCNTs-2.0 were investigated to identify potential interactions between TiO_2_ and the MWCNTs (Fig. 7). In detail, the FTIR spectrum of TiO_2_ (Fig. 7(a)) exhibited a characteristic peak for Ti–O bond vibrations at 547 cm^−1^.^45^ Additionally, peaks were displayed at 3405 and 1631 cm^−1^ corresponding to the stretching and bending vibrations of the O–H group, indicating the presence of adsorbed water molecules on the TiO_2_ surface.^46^ The FTIR spectrum of the MWCNTs showed a characteristic oscillation signal for the C=C bond around 1631 cm^−1^ (Fig. 7(b)).^47^ In the FTIR spectrum of TiO_2_–MWCNTs 2.0 (Fig. 7(c)), peaks also appeared at 3405 and 1631 cm^−1^, suggesting the decoration of TiO_2_ around the MWCNTs.

**Fig. 8:**
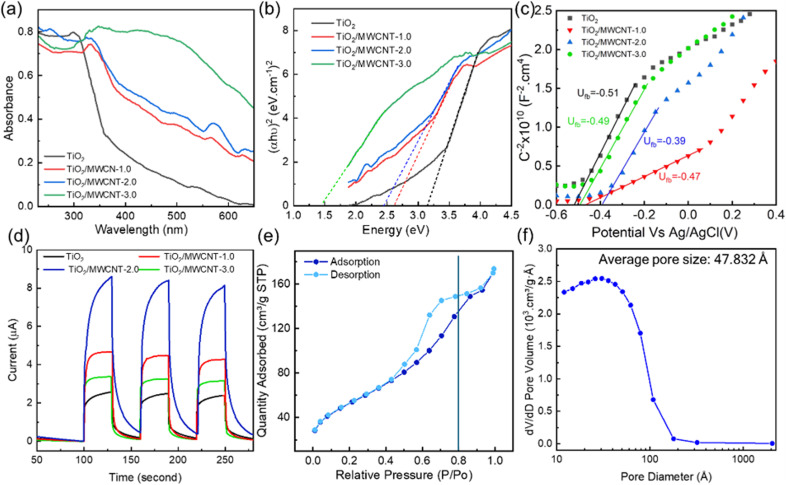
(a) Solid-state UV-vis spectra, (b) Tauc plots for calculation of the band gap, (c) Mott–Schottky plot (d) transient photocurrent responses plots of TiO_2_/MWCNTs nanocomposites synthesized with different MWCNTs contents (0, 1.0%, 2.0%, 3.0%). (c) Mott–Schottky plots of TiO_2_/MWCNTs-1.0 and TiO_2_/MWCNT-2.0, and (e) N_2_ adsorption–desorption isotherms. (f) BHJ adsorption pore-size distribution and isotherms of the TiO_2_/MWCNTs-2.0 nanocomposite.

The UV-vis spectra of pristine TiO_2_ and the TiO_2_/MWCNTs nanocomposites synthesized with varying MWCNT contents (0, 1.0, 2.0, and 3.00 wt%) are presented in Fig. 8(a). The red shift observed in the TiO_2_/MWCNTs nanocomposite samples compared to pristine TiO_2_ indicated a reduction in the electron ionization energy of the nanocomposites, facilitating efficient charge transfer between the TiO_2_ and MWCNTs structure.^16^ The bandgap energy values were calculated from Tauc plots, yielding values of 2.5, 2.4, and 1.5 eV for the nanocomposites with MWCNT contents of 1.0, 2.0, and 3.0 wt%, respectively. These bandgap values were significantly lower than that of pristine TiO_2_, which was 3.1 eV (Fig. 8(b)). The decrease in the bandgaps of the TiO_2_/MWCNTs nanocomposites was attributed to the interaction between TiO_2_ and MWCNTs by forming Ti–O–C bonds. The formation of these chemical bonds creates new energy states within the bandgap of TiO_2_, allowing for longer-wavelength light excitation.^48^ Mott–Schottky (M–S) plots were employed to determine the flat band potential of the TiO_2_/MWCNTs-1.0 and TiO_2_/MWCNTs-2.0 nanocomposites Fig. 8(c). Moreover, a linear regression could be observed in this plot, corresponding to the depletion state of the carriers in the space charge region, as described by eqn (3), characteristic of an n-type semiconductor.^49^ The flat band potentials (*E*_fb_) for TiO_2_, TiO_2_/MWCNTs-1.0, and TiO_2_/MWCNTs-2.0 were approximately –0.51, –0.47, and –0.39 V *vs.* Ag/AgCl, equivalent to –0.31, –0.27, and –0.19 V *vs.* NHE. Based on the formula *E*_VB_ = *E*_CB_ − *E*_g_, the valence band edge (*E*_VB_) values were calculated. Additionally, the valence band potentials for TiO_2_, TiO_2_/MWCNTs-1.0, and TiO_2_/MWCNTs-2.0 were determined to be 2.8, 2.2, and 2.2 eV *vs.* NHE, respectively. The recorded photocurrent response of the pristine TiO_2_ and TiO_2_/MWCNTs nanocomposites showed that the photocurrent induced by the nanocomposites was significantly higher than that of pristine TiO_2_ (Fig. 8(d)). Among them, TiO_2_/MWCNTs-2.0 exhibited the highest photocurrent, three times greater than that of pristine TiO_2_ and twice that of TiO_2_/MWCNTs-1.0. Conversely, TiO_2_/MWCNTs-3.0 displayed the lowest photocurrent response among the nanocomposites. The increased photocurrent signals could be due to the interaction between TiO_2_ and CNTs, which improved the separation rate of photogenerated electrons. However, a drop in the photocurrent value was observed in the TiO_2_/CNT-3.0% composite due to the excess MWCNTs obstructing TiO_2_ light absorption.^50^ This indicates that the MWCNT content in the nanocomposites significantly impacts their photocatalytic properties.

The N_2_ adsorption–desorption isotherms and pore-size distributions of the TiO_2_/MWCNTs-2.0 nanocomposites were obtained and are presented in Fig. 8(e) and (f). The plot shows a type IV adsorption isotherm, indicating capillary condensation in mesopores, with an H1 hysteresis loop appearing at relatively high pressures (0.4 < *P*/*P*_0_ < 1.0). The primary pore diameters were found to be around 4.9 nm. The BET surface area obtained was 189.20 m^2^ g^−1^. Mahdi Kazazi *et al.* prepared a TiO_2_/MWCNT nanocomposite anode material for aqueous RABs using a simple template-free hydrothermal method with pore sizes around 4.02 nm.^51^ In this study, the pore diameter of the material was enhanced by around 4.9 nm. Compared to the previous study,^51^ the surface performance of these nanocomposites was improved. These larger pores in a catalyst could facilitate the adsorption of organic molecules on its surface during photodegradation, thereby boosting its photocatalytic activity. Thus, the addition of MWCNTs can effectively prevent TiO_2_ particle agglomeration and increase the specific surface area.

### 3.5. Photocatalytic of dye degradation

The photocatalytic degradation of MB and RhB dyes was employed to investigate the photocatalyst properties of the modified materials. The effects of many parameters, such as adsorption time, catalyst dosage, pH condition, and type of catalysts, were further investigated.

#### 3.5.1. Effect of the MWCNT contents on dye photodegradation

**Fig. 9:**
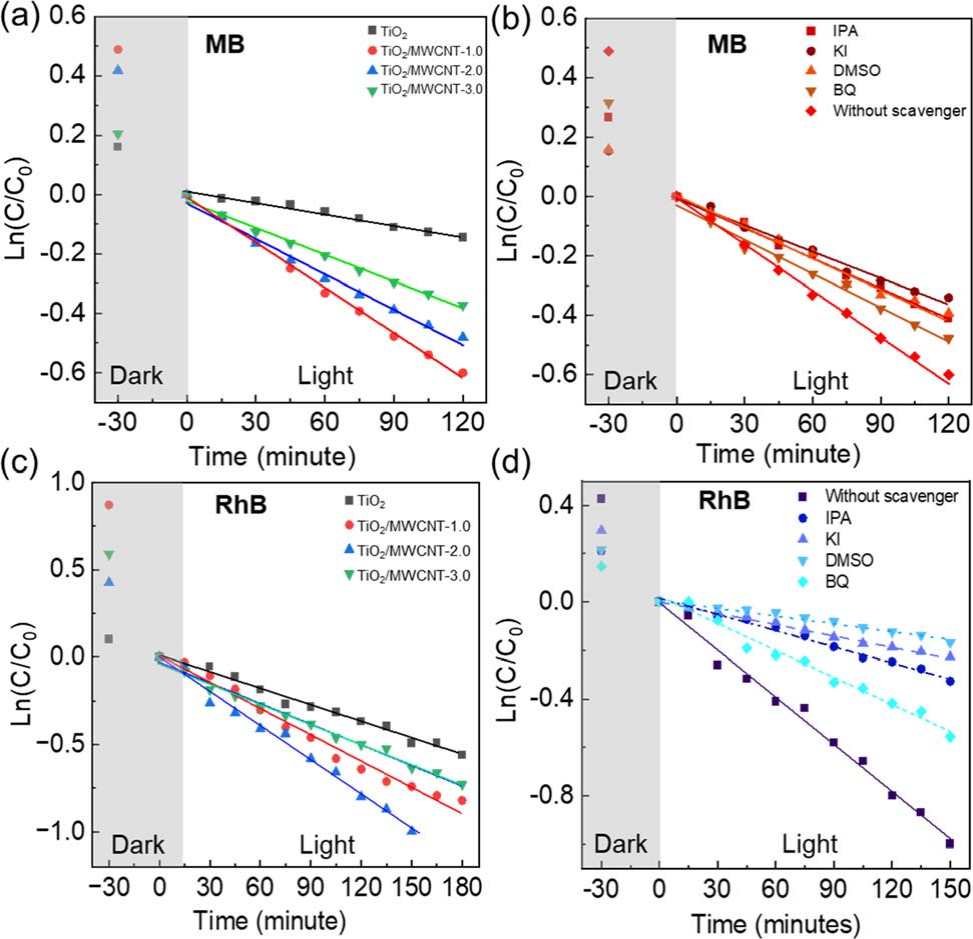
Photocatalytic degradation rate of MB (a) and RhB dyes (c) using TiO_2_/MWCNTs nanocomposite catalysts with different MWCNTs contents (0, 1.0, 2.0, 3.0 wt%). The photocatalytic degradation rates of MB (b) and RhB (d) in the presence of the trapping reagents benzoquinone (BQ), dimethyl sulfoxide (DMSO), isopropyl alcohol (IPA), and potassium iodide (KI) under visible-light irradiation using the TiO_2_/MWCNTs-2.0 nanocomposite.

The dependence of the catalytic activity under visible light on the MWCNT content in the nanocomposite catalysts was further investigated. The kinetics of dye degradation followed a first-order reaction, as evidenced by the linear relationship of ln(*C*_*t*_/*C*_0_) *versus* time (*t*) (Fig. 9). The UV-vis spectra of MB and RhB solutions at different time intervals using pristine TiO_2_ and TiO_2_/MWCNTs nanocomposites are exhibited in Fig. S4 and S5 (ESI†). The rate constants for MB degradation were 0.0051, 0.004, and 0.003 min^−1^ when using TiO_2_/MWCNTs with MWCNT contents of 1.0, 2.0, and 3.0 wt%, respectively (Table S2†). For RhB dye, the rate constants were 0.005, 0.0065, and 0.0039 min^−1^ (Table S2†), respectively. The TiO_2_/MWCNTs-1.0 catalyst demonstrated the highest photocatalytic efficiency for MB photodegradation (Fig. 9(a)). In contrast, TiO_2_/MWCNTs-2.0 exhibited the highest performance for RhB photodegradation (Fig. 9(c)), while TiO_2_/MWCNTs-3.0 showed lower photocatalytic activity for both dyes (Fig. 9(a) and (c)). Fig. S6 and S7 (ESI†) exhibit the change in UV-vis spectra of the MB and RhB solutions during various time intervals when using pristine TiO_2_ and TiO_2_/MWCNTs (with different contents), respectively. It could be seen that the TiO_2_/MWCNTs nanocomposites exhibited significantly enhanced photocatalytic performance compared to pristine TiO_2,_ whose rate constants were 0.0013 and 0.0032 min^−1^ for MB and RhB degradation, respectively. The effects of the irradiation time, pH conditions, and the photodegradation process of dyes using TiO_2_/MWCNTs were also studied (Fig. S8†). The photodegradation rate of RhB dye exhibited a considerable decrease under acidic and alkaline conditions, with corresponding rate constants of 0.0019 and 0.002 min^−1^, respectively, in contrast to 0.0065 min^−1^ under neutral conditions.

The degradation mechanism of MB was previously analyzed based on the bond dissociation energy (BDE) theory by Huang *et al.*^52^ N–CH_3_ was previously dissociated due to having the lowest BDE to form –CH_3_ groups, which could be further oxidized into HCHO or HCOOH. Furthermore, the ˙OH radical has the potential to engage with the C–S^+^ = C functional group in MB. This interaction may result in the cleavage of the C–S^+^ = C bond and the subsequent opening of the aromatic ring structure of phenothiazine. Subsequently, single-ring structures, such as 2,5-diamino benzene sulfonic acid and 4-aminobenzene-1,2-diol, could be formed. Phenyl thiophene is formed from these single-ring structure molecules.^52^ It can be seen from the UV-vis spectra of MB solution that there was a double peak located at 664 and 615 nm, attributed to the monomer and dimers. An increase in the adsorption peak at 615 nm was correlated with more extended time intervals, likely attributable to a higher monomer degradation rate than the dimer. In addition, the decrease in the intensity of the 664 nm peak could be attributed to the *N*-demethylene and the phenothiazine degradation.^53^ For the RhB degradation intermediates, based on the O_2_˙^−^/˙OH generated in the reaction solution, the initial RhB molecules undergo an interruption step, such as N-de-ethylation, chromophore breaking, opening-ring, and mineralization. These primary intermediates from the degradation of RhB could be benzoic acid, succinic acid, 2-hydroxy-pentane dioic acid, adipic acid, 3-hydroxybenzoic acid, phthalic acid, and terephthalic acid.^54^

The enhanced photocatalytic performance of the TiO_2_/MWCNTs nanocomposite was attributed to the formation of Ti–O–C bonds, as evidenced by the XPS results. These bonds generate new electronic energy states between the valence band (VB) and conduction band (CB) of TiO_2_. This increases the number of photogenerated holes (h^+^) and electrons (e^−^) because less energy from the incident light is required to separate the electrons and holes. Additionally, MWCNTs, known for their high work function and conductivity, act as electron sinks that reduce the h^+^ and e^−^ recombination rate. This increase in the lifetime of h^+^ and e^−^ significantly improves the photodegradation rate of dye molecules.^55^

The photodegradation of dyes involves the oxidation of free radical species, such as excited electrons (e^−^), photoexcited holes (h^+^), hydroxyl radicals (˙OH), and superoxide radicals (˙O^2−^).^56^ The roles of these free radical species in the photodegradation mechanism of dyes with the TiO_2_/MWCNTs-2.0 nanocomposite catalysts were explored using potassium iodide (KI), isopropanol (IPA), benzoquinone (BQ), and DMSO as the trapping reagents of h^+^, ˙OH, ˙O^2−^, and e^−^, respectively. The UV-vis spectra of RhB solutions at the adsorption time intervals using TiO_2_/MWCNTs-2.0 nanocomposites in the presence of different radical trapping agents are shown in Fig. S9.† It was found that the degradation rate in the presence of the trapping reagents DMSO, KI, IPA, and BQ was reduced by 83.1%, 76.0%, 65.9%, and 36.7% for RhB dye (Fig. 9(b)), while that for MB dye corresponded to 32.7%, 41.1%, 32.1%, and 24.6% (Fig. 9(d)) in comparison to without using trapping reagents. Therefore, it could be assumed that RhB photodegradation occurred through the contribution of free radicals in the order of e^−^ > h^+^ > ˙OH > ˙O^2−^ and the ability to capture radicals in the case of MB dye corresponded to h^+^ > e^−^ > ˙OH > ˙O^2−^.

The mechanism of organic compound degradation using the TiO_2_/MWCNTs photocatalyst suggested by Chen *et al.* is based on electron transfer from the TiO_2_ to CNTs.^16^ Furthermore, in the presence of hydrogen ions and water molecules, the decomposition of MB and RhB could be described as follows (eqn (9)–(19)):

9 Dye + *hv* → dye* + e^−^

10 Dye* + TiO_2_ → TiO_2_ (e_CB_^−^) + dye

11 Dye* → dye + h_VB_^+^

12 TiO_2_ + *hv* → TiO_2_ (e_CB_^−^) + h_VB_^+^

13 TiO_2_ (e_CB_^−^) + MWCNTs → TiO_2_ + MWCNTs (e^−^)

14 MWCNTs (e^−^) + O_2_ → MWCNTs + ˙O_2_^−^

15 ˙O_2_^−^ + H_2_O → HO_2_^−^ + ˙OH

16 HO_2_^−^ + H^+^ → H_2_O_2_

17 h_VB_^+^ + 2OH^−^ → ˙OH +0.5O_2_

18 ˙OH + dye → degradation product

19 h_VB_^+^ + dye → degradation product

In reaction (14), oxygen reacts with electrons on MWCNTs to form free radicals ˙O_2_^−^, leading to the formation of ˙OH radicals (reaction (15)), which catalyzes the decomposition reaction of RhB (reaction (18)). If ˙O_2_^−^ radicals are limited and little formed, the efficiency of the RhB degradation reaction will be significantly reduced. Previous studies also showed that superoxide radicals ˙O_2_^−^ and HO_2_^−^ have a very strong activity that can degrade the aromatic ring structure of some organic compounds.^57^

#### 3.5.2. Cyclic performance of TiO_2_/MWCNTs

The recyclability of photocatalysts is crucial because it allows for their repeated use and significantly reduces costs. The recyclability of photocatalysts containing 2.0% MWCNTs was evaluated over six cycles, and the photodegradation efficiency was calculated using eqn (1). Fig. S10 (ESI†) presents the degradation efficiency across six cycles. In the first two cycles, the degradation efficiencies were 55.3% and 47.1%. In the third cycle, the efficiency remained nearly unchanged at 47.1%. By the fourth cycle, there was a slight decline to 45.2%. The efficiency dropped to 40.0% in the fifth cycle, and by the final cycle, the photocatalyst's recyclability had decreased to 33.3%.

The thermal properties of the TiO_2_/MWCNTs nanocomposite before and after the photocatalyst recyclability test were evaluated using thermogravimetric analysis (TGA). The investigations were performed from room temperature to 800 °C. The TGA curves of the TiO_2_/MWCNTs composites heated in air are depicted in Fig. S11.† The observed weight losses at 40 °C to 200 °C corresponded to the evaporation of residual solvents and water. At a temperature of 200 °C, complete removal of the water and organic precursors occurred, resulting in residual masses of 96.67% and 96.98% of the initial weight of the TiO_2_/MWCNT-2.0 sample after and before the photocatalyst recyclability test, respectively. The sharp weight loss at 200 °C signaled the onset of MWCNTs oxidation, with the activation energy for this process being influenced by factors such as the number of walls, defects, and impurities present in the MWCNTs.^58^ The thermogravimetric analysis also revealed a two-step weight loss pattern: the first stage, at lower temperatures, was due to the loss of solvents and water, while the second stage, at higher temperatures, corresponded to the oxidation of MWCNTs. The TiO_2_ content in the composites was determined through TGA, assuming complete MWCNTs oxidation at 480 °C. Following six cycles, the TiO_2_/MWCNTs-2.0 sample exhibited mass percentages of 90.17% and 91.99% post- and pre-photocatalyst recyclability testing (Fig. S11†), respectively, implying the recyclability of the photocatalyst. After six cycles of testing in the photodegradation reaction, the changes in the TiO_2_/MWCNTs nanocomposite structure were investigated based on the FTIR spectrum (Fig. S12†). It was observed that the intensity of the characteristic peaks attributed to the vibrations of O–H, C=H, CH_2_, and Ti–O groups remarkably increased after six cycles of the photocatalytic performance studies, indicating the degradation of the TiO_2_/MWCNTs nanocomposite.

The efficacy of the as-prepared TiO_2_/MWCNTs was also compared with that of commercial TiO_2_ under similar conditions. Experiments were conducted to assess the photodegradation of dyes. The findings indicated that the TiO_2_/MWCNTs nanocomposite outperformed commercial TiO_2,_ as depicted in Fig. S13.†

#### 3.5.3. Photodegradation mechanism

TiO_2_ is a well-known semiconductor with a band gap larger than 3.0 eV, with the valence band attributed to the occupied O 2p orbital and the conduction band involving the unoccupied Ti 3d orbital, making TiO_2_ primarily active under UV light. When exposed to UV light, electrons in the valence band of TiO_2_ are excited into the conduction band, forming photogenerated electron–hole (e^−^/h^+^) pairs. The lifetime of these e^−^/h^+^ pairs is only at the nanosecond level, leading to their rapid recombination and limiting their participation in photocatalytic reactions.^55^ Integrating MWCNTs into the TiO_2_ matrix significantly enhanced the photocatalytic performance. The TiO_2_/MWCNTs nanocomposites exhibited higher photocatalytic degradation efficiency for MB and RhB dyes, which could be attributed to the formation of new energy bands within the TiO_2_ band gap, created by the formation of new Ti–O–C bonds in the TiO_2_ structure. This modification narrows the band gap of the nanocomposite materials, leading to greater absorption in the visible-light spectral range, which accounts for a dominant part of sunlight.

Another advantage of MWCNTs is their role as an electron sink. Due to the high conductivity of MWCNTs, photogenerated electrons can easily transfer to the carbon nanotubes, which limits the interaction between electrons and holes, slowing down the charge-carrier recombination rate. This behavior was similarly observed in the case of MWCNTs, which also served as electron-trap centers to slow the recombination rate of electron–hole e^−^/h^+^ pairs.^16^ MWCNTs possess a great number of active sites, resulting in a strong affinity for the targeted contaminant molecules on the surface of the photocatalyst.^59^ The positively charged non-metal and MWCNTs also promote photocatalytic activity by increasing the rate of e-transfer to dissolved oxygen molecules by generating highly reactive superoxide ions radicals (˙O_2_^−^), which oxidize the pollutants. The h^+^ may oxidize OH^−^ or H_2_O to form the most potent and non-selective hydroxyl radical (˙OH), which can degrade a wide range of organic dyes and biomolecules.

## 4 Conclusion

In the study, we successfully synthesized TiO_2_/MWCNTs nanomaterials using a hydrothermal method and assessed their photocatalytic performance in dye decomposition under simulated sunlight. The TiO_2_/MWCNTs nanomaterials exhibited a pore size of 4.9 nm and a BET surface area of 189.20 m^2^ g^−1^, representing an increase over previous studies. The characterization studies showed that TiO_2_ nanoparticles were successfully decorated on the MWCNTs surface, providing sufficient active sites on the photocatalyst surface. Additionally, incorporating CNTs reduced the bandgap from 3.1 eV to 2.5 eV with 1.0% MWCNTs and 2.4 eV in 2% MWCNTs and suppressed electron–hole recombination. The effects of the adsorption time, the percentage of MWCNTs in the nanomaterial, and the catalyst dose on the photocatalytic properties of pure TiO_2_ and TiO_2_/MWCNTs were carefully studied. TiO_2_/MWCNTs-2.0 was more effective for RhB degradation, boosting a rate constant of 0.0065 min^−1^. These findings suggested that the TiO_2_/MWCNTs nanocomposites had significantly enhanced photocatalytic performance compared to TiO_2_, with rate constants of 0.0013 min^−1^ for MB and 0.0032 min^−1^ for RhB degradation. This modified approach can contribute to a novel approach for the rapid synthesis of nanocomposites and can be extended to the degradation of other organic pollutants.

## Data availability

The data used to support the findings of this study are included in the article.

## Author contributions

Conceptualization: Nhu-Bao Trinh, Thu Anh Nguyen, Sy Van Vu. Methodology and analysis: Nhu-Bao Trinh, Sy Van Vu, Hong-Gam Thi Vo, In Park, Tien Nu Hoang Lo, Thu Anh Nguyen, and Khuong Quoc Vo. Investigation: Nhu-Bao Trinh, Hong-Gam Vo Thi, In Park, Tien Nu Hoang Lo, and Thu Anh Nguyen. Writing – original draft preparation: Nhu-Bao Trinh, Thu Anh Nguyen, Khuong Quoc Vo. Writing – review and editing: Nhu-Bao Trinh, Sy Van Vu, Thu Anh Nguyen, Hong-Gam Thi Vo, and Khuong Quoc Vo. Supervision: Nhu-Bao Trinh, In Park, Khuong Quoc Vo. All authors reviewed the manuscript.

## Abbreviations

DI: Deionized
MWCNTs: Multiwalled carbon nanotubes
MB: Methylene blue
RhB: Rhodamine B
TiO_2_: Titanium dioxide
TiO_2_/MWCNTs: Titanium dioxide-decorated MWCNTs

## Conflicts of interest

There are no conflicts to declare.

## Supplementary Material

RA-014-D4RA05899B-s001
