# Supplementary material for: Modified hydrothermal method for synthesizing titanium dioxide-decorated multiwalled carbon nanotube nanocomposites for the solar-driven photocatalytic degradation of dyes
Source: RSC Adv. 2024 Oct 25;14(46):34037–50. doi: 10.1039/d4ra05899b (PMC11506254; doi:10.1039/d4ra05899b)
Supplement: RA-014-D4RA05899B-s001 [file RA-014-D4RA05899B-s001.pdf]

Supplementary Information (SI) for RSC Advances.
This journal is © The Royal Society of Chemistry 2024

SUPPLEMENTARY INFORMATION

for

MODIFIED HYDROTHERMAL METHOD FOR SYNTHESIZING THE TITANIUM DIOXIDE-DECORATED MULTIWALLED CARBON NANOTUBES NANOCOMPOSITE FOR PHOTOCATALYTIC DEGRADATION OF DYES

Nhu- Bao Trinh[1,2], Thu Anh Nguyen[1,2], Sy Van Vu[1,2], Hong- Gam Thi Vo[1, 2], Tien Nu Hoang Lo[3], In Park[3,4], Khuong Quoc Vo[1,2*].

[1] Faculty of Chemistry, Ho Chi Minh City University of Science, Vietnam National University, Ho Chi Minh City, 227 Nguyen Van Cu Street, Ward 4, District 5, Ho Chi Minh City 70000, Vietnam.

[2] Vietnam National University, Ho Chi Minh City, Vietnam.

[3] Research Institute of Clean Manufacturing System, Korea Institute of Industrial Technology (KITECH), 89 Yangdaegiro-gil, Ipjang-myeon, Cheonan 31056, South Korea.

[4] KITECH school, University of Science and Technology (UST), 176 Gajeong-dong, Yuseong-gu, Daejeon 34113, South Korea.

* Corresponding author

Email: vqkhuong@hcmus.edu.vn

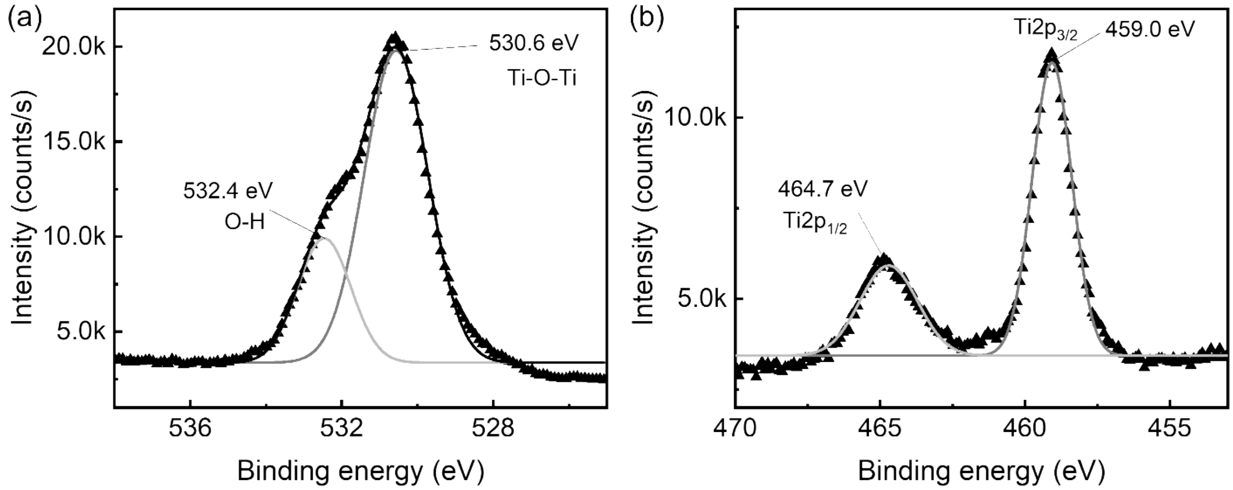

Figure S1. (a) High-resolution O1s XPS spectra (b) Ti2p XPS spectra of bare TiO_2.

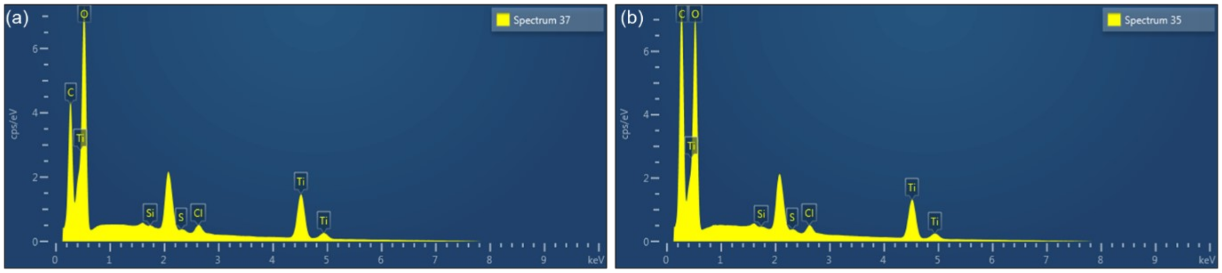

Figure S2. EDS spectra of TiO_2/MWCNT nanocomposites with the MWCNTs contents of (a) 1.0 and (b) 2.0 wt.%.

Table S1. Corresponding elements composition of EDS spectrum of (a) TiO_2/MWCNTs nanocomposites with the sample of TiO_2/MWCNTs of (a) 1.0 and (b) 2.0 wt. %.

**Sample Name**	**Wt./At. (%)**	**Carbon C K**	**Oxygen O K**	**Titanium Ti K**	**Chlor Cl K**	**Sulfur S K**	**Silic S K**	**Total**
TiO_2-	Wt.%	14.06	32.69	52.10	0.94	0.09	0.12	100.00
CNT-1%	At.%	27.01	47.13	25.09	0.61	0.07	0.10	100.00
TiO_2-	Wt.%	22.41	29.07	47.42	0.91	0.12	0.07	100.00
CNT-2%	At.%	39.66	38.62	21.04	0.55	0.08	0.06	100.00

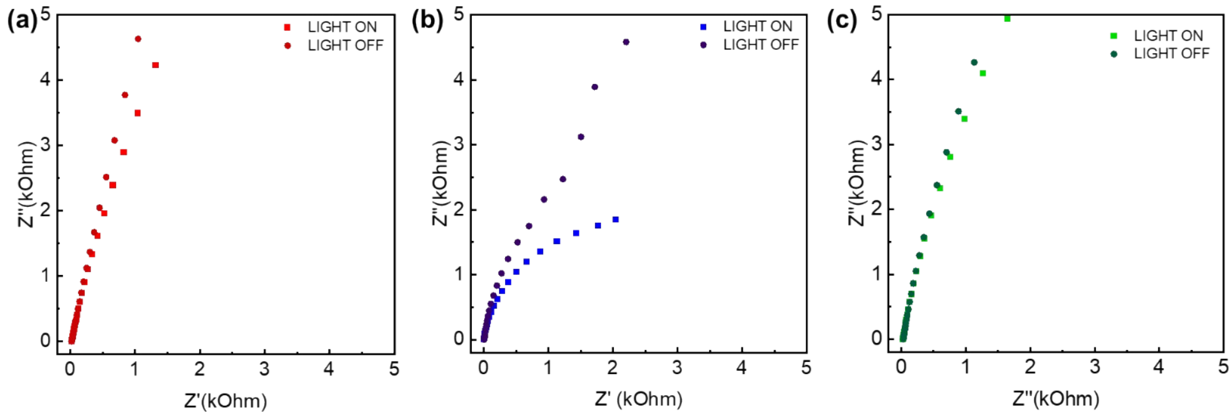

Figure S3. EIS of the TiO_2/MWNCT with different MWCNTs contents varies from 1.0, 2.0, and 3.0% investigated under light ON/OFF conditions.

Adsorption time

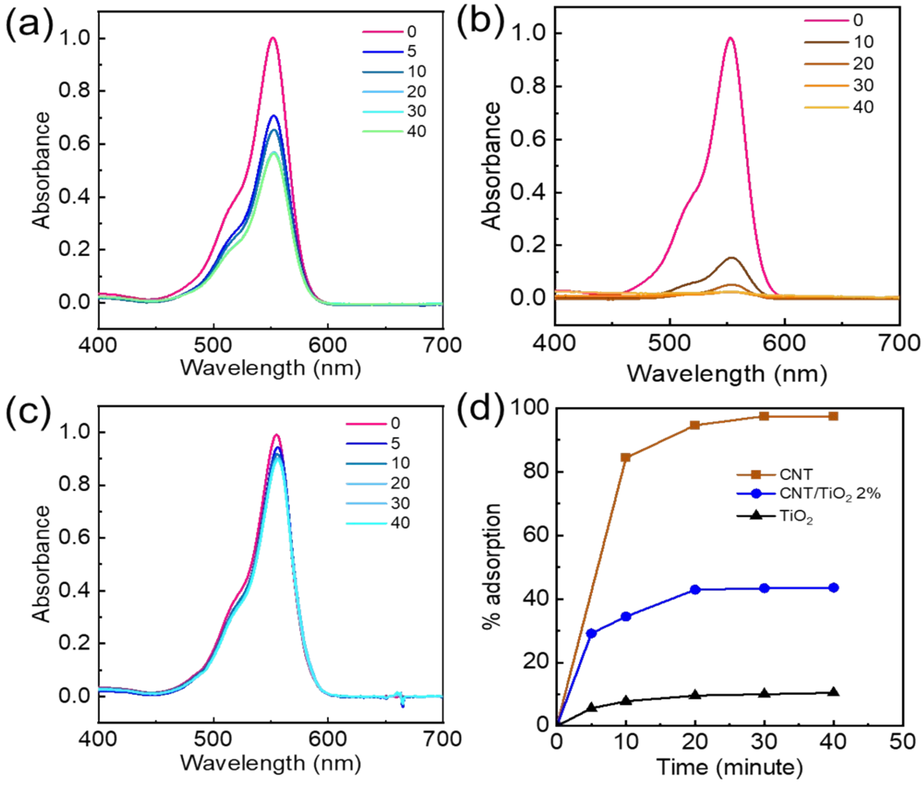

Figure S4. The UV-Vis spectra of RhB solution at the adsorption time intervals of (a) TiO_2/MWCNT-2.0 (b), MWCNTs, (c) pristine TiO_2, (d) the plot present the adsorption percentage along with time at Temp (30 °C).

It is vital to investigate the adsorption time of the photocatalyst to achieve good efficiency in enhanced dye degradation. Figure S4 (a)-(c) shows the adsorption spectra of RhB on TiO_2/MWCNT-2.0, MWCNTs, and pristine TiO_2, respectively. These spectra show that the adsorption capacity rose during the first ten minutes, then the adsorption rate slowed after 20 minutes and remained unchanged when stirred for 30 minutes (Figure S4(d)). The 30 minutes was chosen as the adsorption time needed to reach the adsorption-desorption equilibrium before illumination. It can be explained that TiO_2/CNTs have many available adsorption sites on the

adsorbent surface, leading to a high adsorption rate [110]. As time progresses, these sites become occupied, and fewer sites are available for further adsorption, resulting in a slower rate. Therefore, in the following photocatalytic experiments, the reaction solution was mixed with the photocatalyst for 30 minutes before activating the light.

Catalyst dosage

Photocatalyst dosage is a crucial factor influencing the photocatalytic degradation process [48]. Figure S5 shows the time dependence of relative concentration Ln(C/C_o) of MB in the solution at different photocatalytic dosages of 0.2, 0.4, and 0.6 g/L. The results indicated that using 0.4 g/L of the catalyst with a rate constant of 6.49×10^{-3} s^{-1} can significantly boost the degradation of MB in water. This catalyst dosage could achieve a rate ten times higher than that observed without the catalyst, with a rate constant of 0.618×10^{-3}. We also found that the degradation rate concomitantly increased with the catalyst dosage, rising from 0.20 to 0.40 g/L. That could be due to the increase in active site density on the catalyst [20]. More active sites may lead to more efficient adsorption and activation of reactants, thereby enhancing the degradation rate. However, increasing the catalyst dosage to 0.60 g/L conversely reduced the decolorization rate of MB. This reduction was likely due to excessive catalysts, which limited the exposure of the incident light and the catalyst [20].

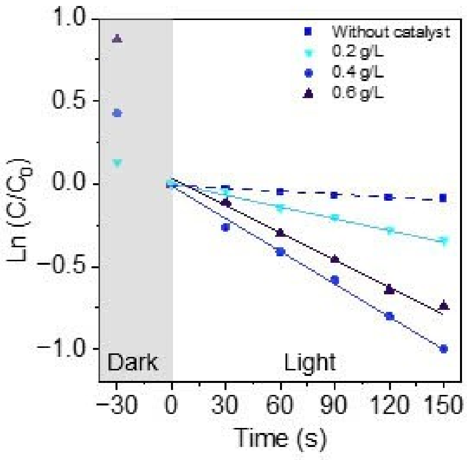

Sample name	Linear equation (MB)
Without catalyst	$Y = -0.618.10^{-3} X - 0.908.10^{-2}$
0.2 g/L	$Y = -2.38.10^{-3} X + 0.907.10^{-2}$
0.4 g/L	$Y = -6.49.10^{-3} X + 2.80.10^{-2}$
0.6 g/L	$Y = -5.20.10^{-3} X + 1.55.10^{-2}$

Figure S5. The photocatalytic degradation rate of methylene blue (MB) at different catalyst dosages from 0.20 to 0.60 g/L.

Effect of pH

To investigate the effect of pH on the photocatalytic degradation process, dye decomposition was studied in acidic (pH 3), neutral (pH 7), and alkaline (pH 10) environments (see Figure S6). The results showed that the photodegradation rate of RhB dye was significantly lower under acidic and alkaline conditions, with rate constants of 0.0019 min^{-1} and 0.002 min^{-1}, respectively, compared to 0.0065 min^{-1} under neutral conditions (Figure S6(c)).

$$TiOH + H^{+} \rightleftarrows TiOH_{2}^{+} \tag{18}$$

$$TiOH + OH^{-} \rightleftarrows TiO^{-} + H_{2}O \tag{19}$$

TiO_2 is hydrated to form Ti–OH groups on the surface. In acidic pH, Ti-OH takes up H^+ ions and acquires a positive charge, whereas, in basic pH, Ti-OH loses protons to OH^- ions and acquires a negative charge [117]. MB is a cationic dye and, therefore, will preferentially adsorb on negatively charged TiO_2. However, the dye becomes positively charged in acidic solutions while remaining neutral. molecule in basic solutions. In acidic solutions, due to electrostatic repulsion, the rate of adsorption of MB on TiO2 decreases, lowering the rate of degradation.

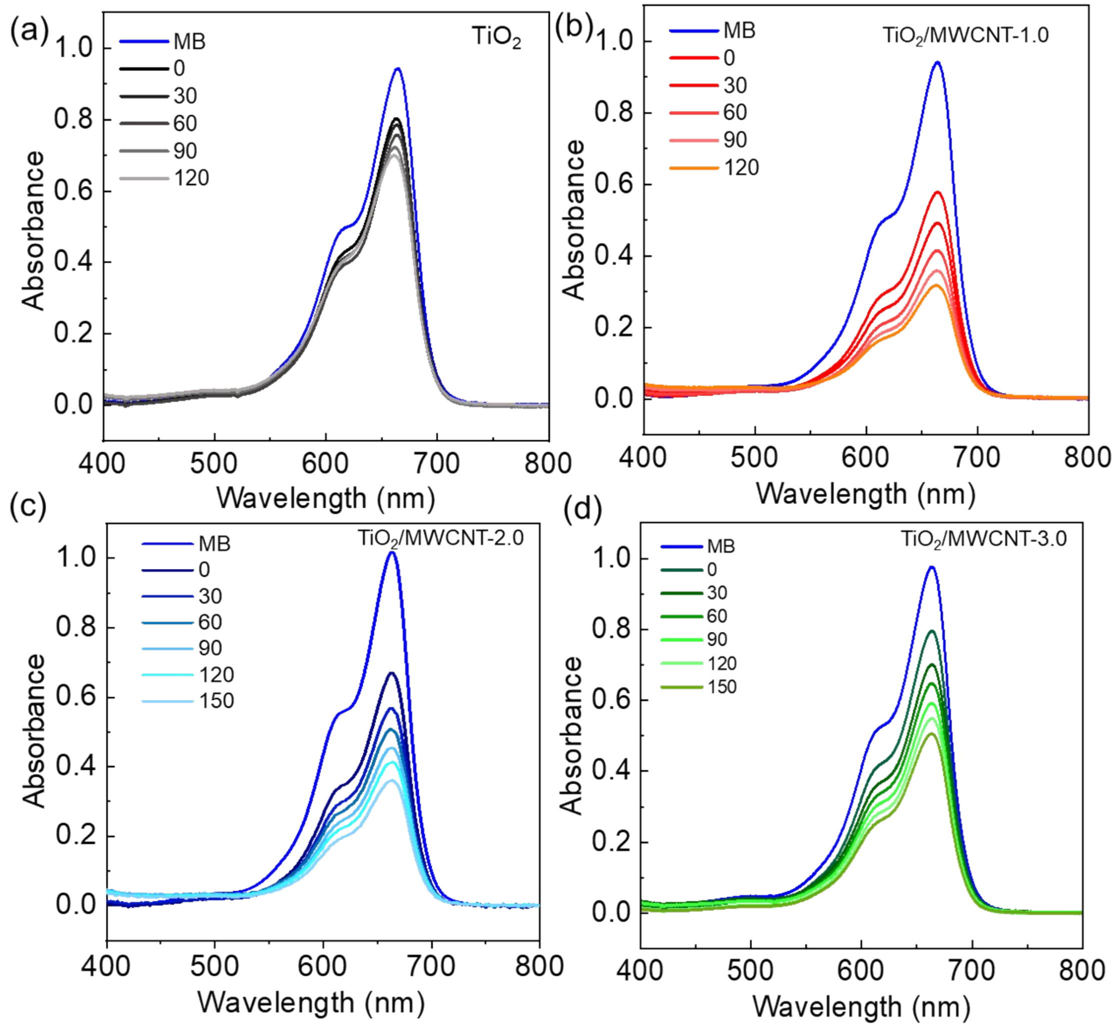

Figure S6. The UV-Vis spectra of MB solution at various adsorption time intervals using (a) pristine TiO_2 and TiO_2/MWCNTs nanocomposite catalyst synthesized with different content MWCNT (b) 1.0, (c) 2.0 (d) 3.0%

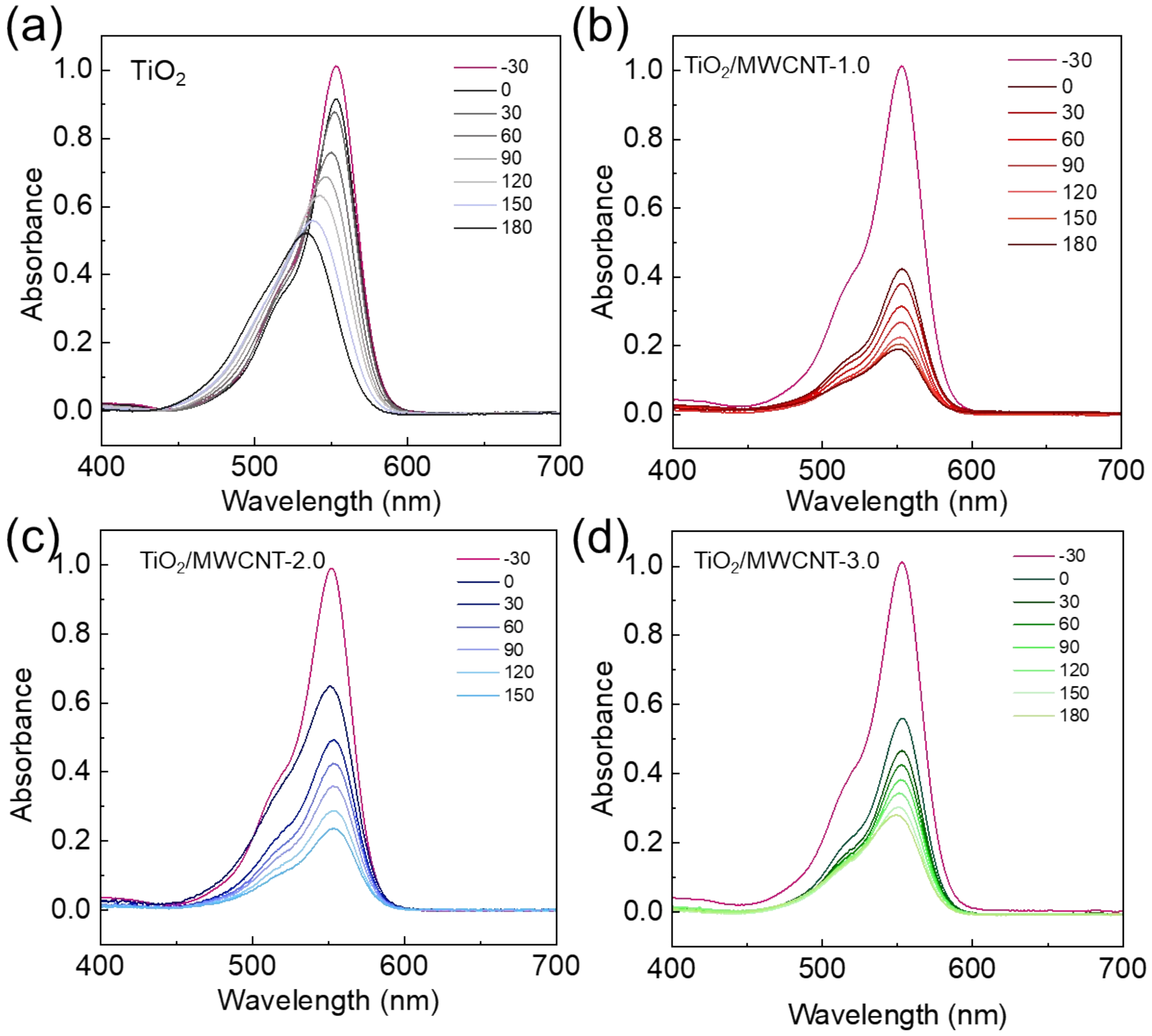

Figure S7. The UV-Vis spectra of RhB solution at different adsorption time intervals with (a) pristine TiO_2 and TiO_2/MWCNTs nanocomposite catalyst synthesized with different content MWCNT (b) 1.0 %, (c) 2.0% (d) 3.0 %

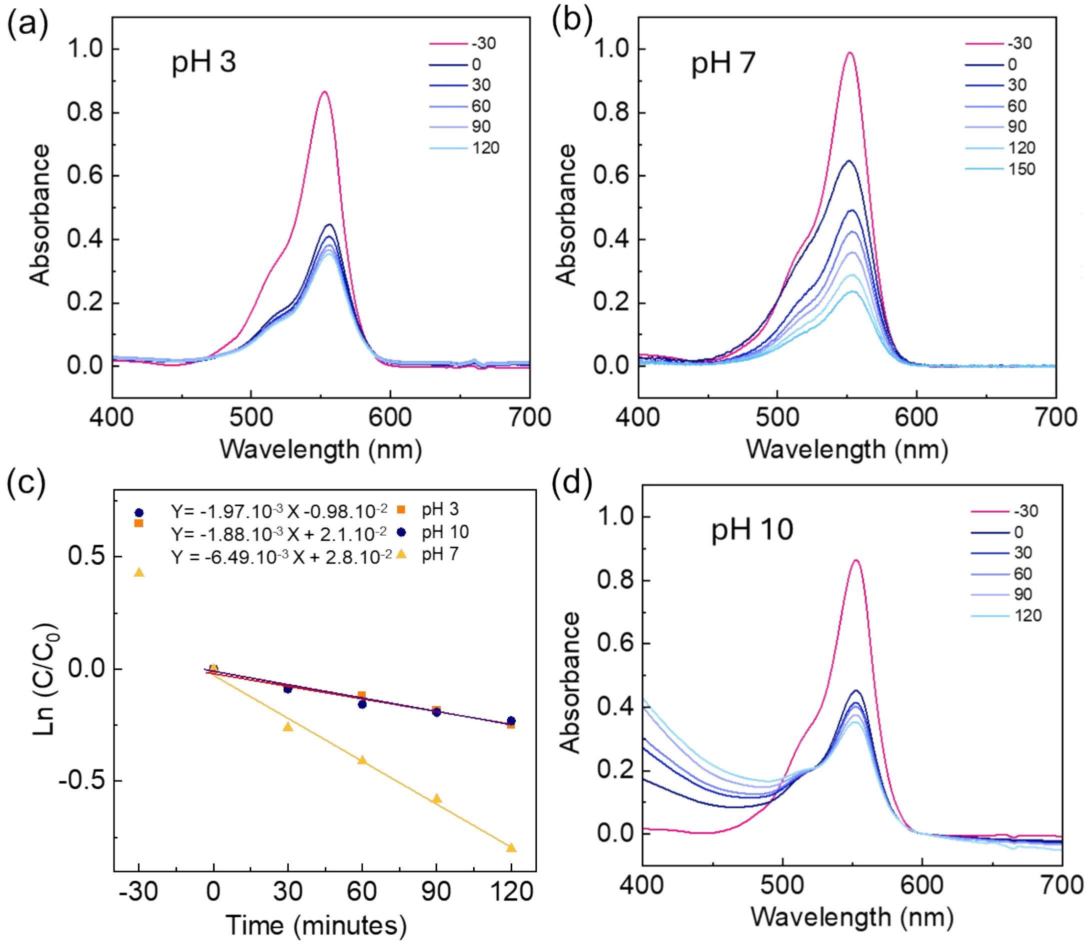

Figure S8. The UV-Vis spectra of RhB solution at different time irradiation using 0.4 g/L TiO_2/MWCNTs-2.0 under pH conditions (a) pH 3, (b) pH 7, (d) pH 10. (c) Linear regression line between Ln(C/C_o) and the reaction times at various pH conditions of 3, 7, and 10.

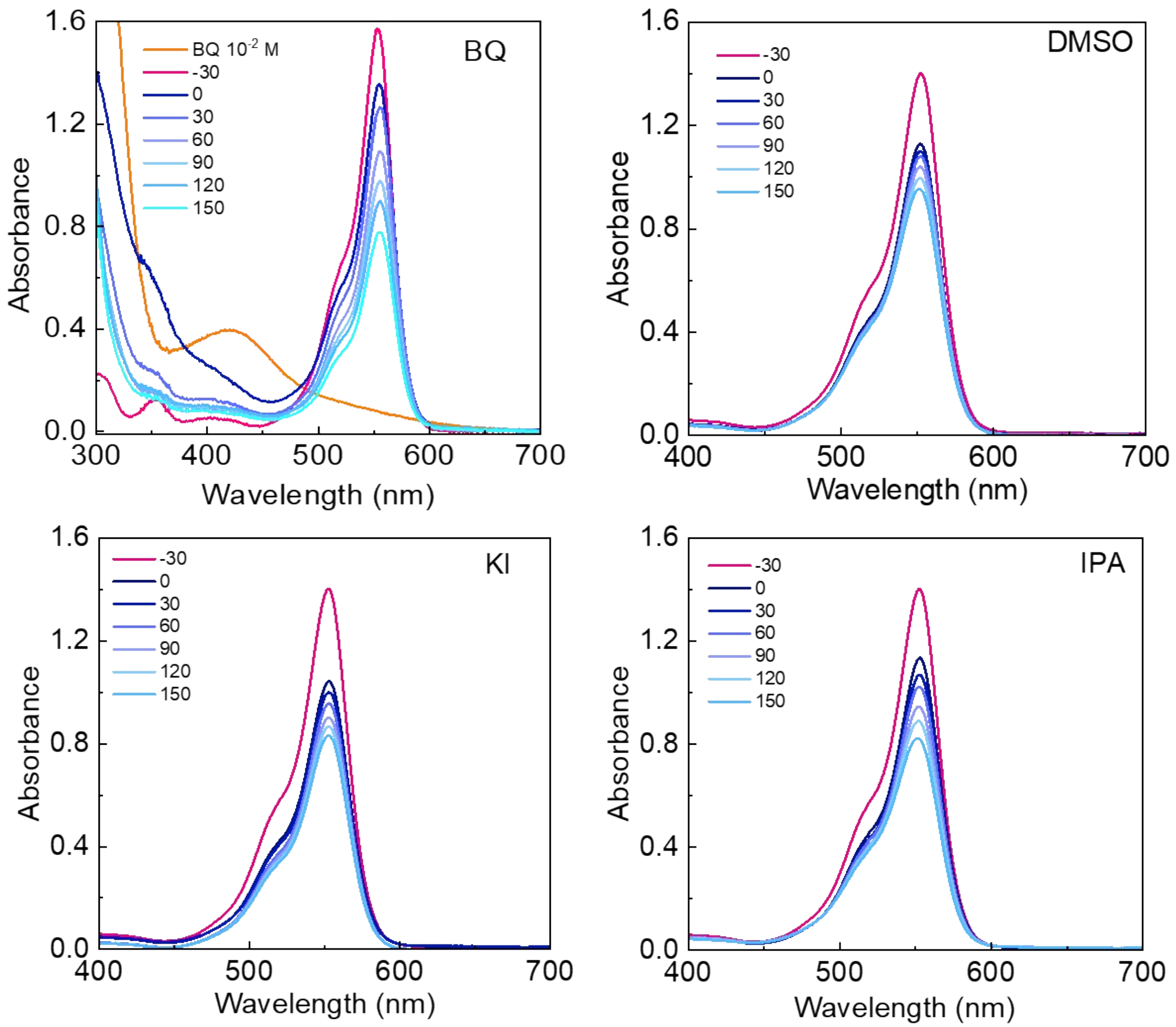

Figure S9. The change of RhB UV-Vis spectra at various adsorption time intervals tested with TiO_2/MWCNTs-2.0 nanocomposites in the presence of different radical trapping agents (a) BQ, (b) DMSO, (c) KI, (d) IPA.

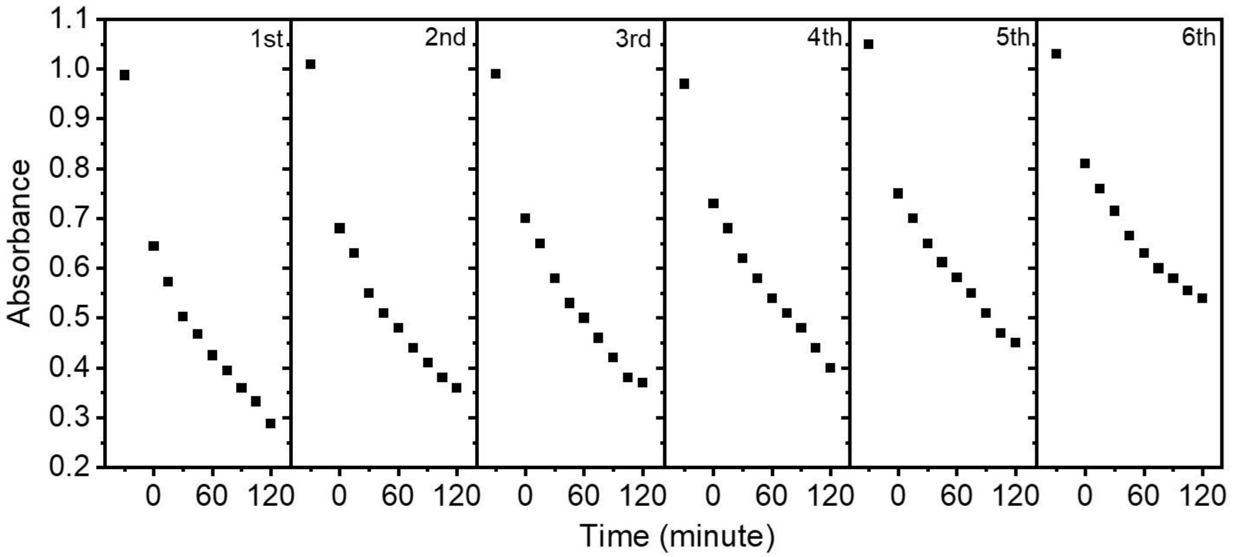

Figure S10. Cyclic runs of the photo-degradation of RhB using TiO_2/MWCNTs-2.0 under sunlight irradiation through six cycles.

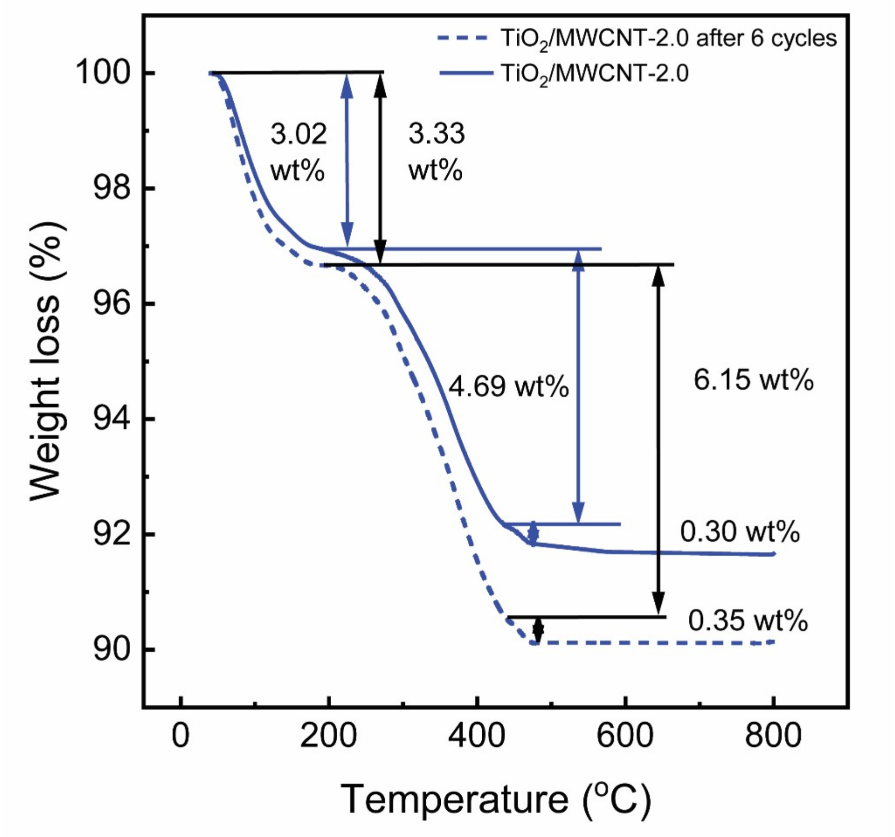

Figure S11. TGA results for TiO_2/MWCNTs-2.0 before and after the photocatalyst recyclability test.

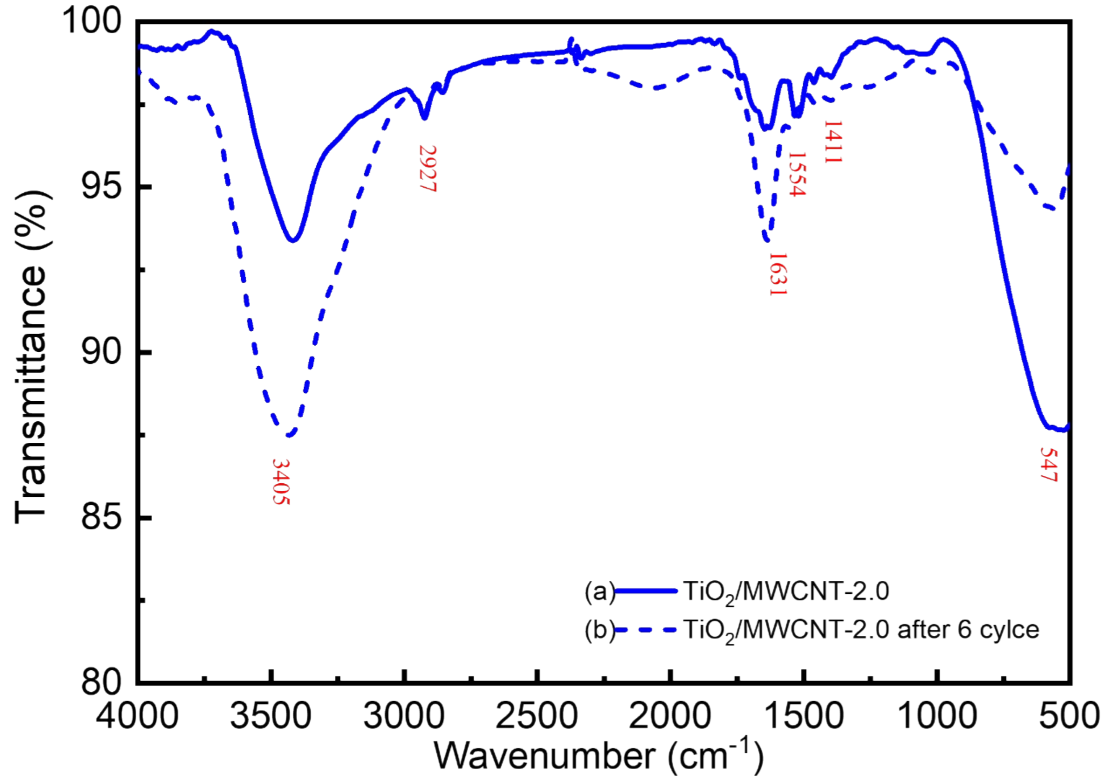

Figure S12. FTIR spectra of the as-prepared TiO_2/MWCNTs-2.0 nanocomposite for the first time and after six times use in the photodegradation process.

Compared to the commercial TiO_2

Typically, 40.0 mg of commercial TiO_2 (Merk, Germany, >99.0%) CAS: 13463-67-7 was introduced into 100 mL of a 5.0 mg/L aqueous dye solution. Subsequently, the solution was stirred at 300 rpm in darkness for 30 minutes to establish adsorption/desorption equilibrium, followed by irradiation to initiate the dye degradation process. After each interval, a 5.0 mL aliquot was extracted and centrifuged at 6000 rpm to eliminate the catalyst. The dye concentration was quantified using UV-Vis spectroscopy at 664 and 554 nm. Photodegradation efficiency was determined using equation (1), detailed in the experimental section. It can be seen from Figure S13 that the linear regression of MB was $y = -2.73\bullet10^{-4}x - 2.19\bullet10^{-3}$, which possessed a rate constant value that is remarkably lower than the one using 0.4 g/L TiO_2/MWCNT-3.0 nanocomposite catalysts ($-3.83\bullet10^{-3}$ in the linear equation of $y=-3.83\bullet10^{-3}x + 2.78\bullet10^{-2}$, Table S2, supporting information part). Similar observation results were obtained when investigating RhB, and Figure S13 shows that the rate constant was $-1{,}86\bullet10^{-4}$, which is significantly lower than the sample of TiO_2/MWCNT-2.0, which had a rate constant of $-6{,}49\bullet10^{-3}$ (Table S2, supporting information part). It can be inferred that the as-prepared nanocomposite catalyst has a better effectiveness of MB and RhB than the commercial TiO_2.

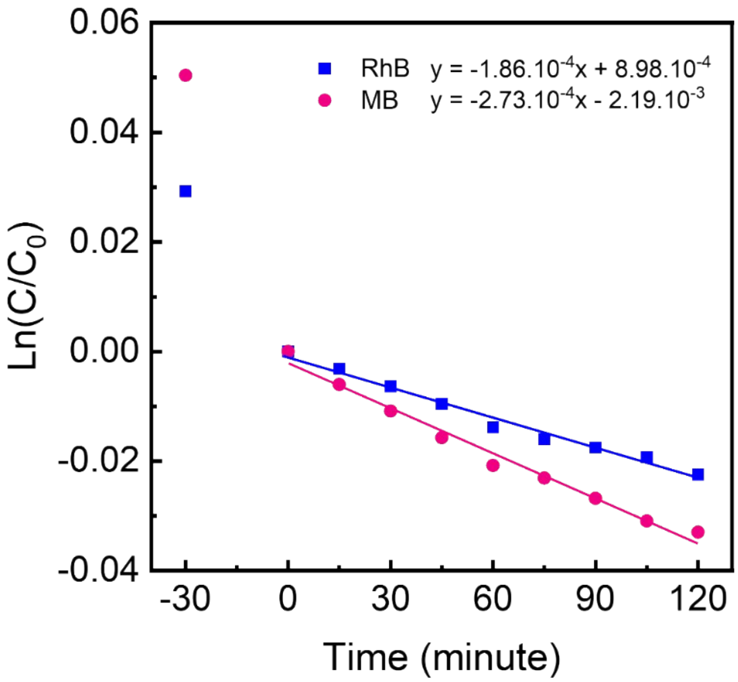

Figure S13. The photocatalytic degradation rate of Rhodamine B and methylene blue (MB) with the TiO_2 commercial catalyst.

Table S2. The linear fit equations for the photodegradation of MB (methylene blue) and RhB (rhodamine B) dyes using TiO_2/MWCNT nanocomposite catalysts with different MWCNT contents (0, 1.0, 2.0, 3.0 wt%). The linear fit equations for the photodegradation of MB (methylene blue) and RhB (rhodamine B) dyes using TiO_2/MWCNT-2.0 nanocomposite in the presence of trapping reagents such as benzoquinone (BQ), dimethyl sulfoxide (DMSO), isopropyl alcohol (IPA), and potassium iodide (KI).

**Sample name**	**Linear equation (RhB)**	**Linear equation (MB)**
TiO_2	$Y = -3.16.10^{-3} X + 10.1.10^{-2}$	$Y = -5.0810^{-3} X + 9.21.10^{-2}$
TiO_2/MWCNT-1.0	$Y = -5.0210^{-3} X + 8.39.10^{-2}$	$Y = -3.45.10^{-3} X + 0.42.10^{-2}$
TiO_2/MWCNT-2.0	$Y = -6.49.10^{-3} X + 2.80.10^{-2}$	$Y = -2.99.10^{-3} X + 5.52.10^{-2}$
TiO_2/MWCNT-3.0	$Y = -3.87.10^{-3} X + 3.56.10^{-2}$	$Y = -3.83.10^{-3} X + 2.78.10^{-2}$

**Radical scavenger**	**Linear equation (RhB)**	**Linear equation (MB)**
Without RS	$Y = -6.49.10^{-3} X + 2.80.10^{-2}$	$Y = -5.0810^{-3} X + 9.21.10^{-2}$
IPA	$Y = -2.21.10^{-3} X + 15.7.10^{-2}$	$Y = -3.45.10^{-3} X + 0.42.10^{-2}$
KI	$Y = -1.56.10^{-3} X + 2.10.10^{-2}$	$Y = -2.99.10^{-3} X + 5.52.10^{-2}$
BQ	$Y = -4.11.10^{-3} X + 9.88.10^{-2}$	$Y = -3.83.10^{-3} X + 2.78.10^{-2}$
DMSO	$Y = -1.10.10^{-3} X + 2.13.10^{-2}$	$Y = -3.42.10^{-3} X + 0.73.10^{-2}$
